# Integrated transcriptome and proteome analysis provides insight into chilling-induced dormancy breaking in *Chimonanthus praecox*

**DOI:** 10.1038/s41438-020-00421-x

**Published:** 2020-12-01

**Authors:** Zhineng Li, Ning Liu, Wei Zhang, Chunyu Wu, Yingjie Jiang, Jing Ma, Mingyang Li, Shunzhao Sui

**Affiliations:** Key Laboratory of Horticulture Science for Southern Mountains Regions, Ministry of Education, Chongqing Engineering Research Center for Floriculture, College of Horticulture and Landscape Architecture, Southwest University, 400715 Chongqing, China

**Keywords:** Abiotic, Transcriptomics

## Abstract

Chilling has a critical role in the growth and development of perennial plants. The chilling requirement (CR) for dormancy breaking largely depends on the species. However, global warming is expected to negatively affect chilling accumulation and dormancy release in a wide range of perennial plants. Here, we used *Chimonanthus praecox* as a model to investigate the CR for dormancy breaking under natural and artificial conditions. We determined the minimum CR (570 chill units, CU) needed for chilling-induced dormancy breaking and analyzed the transcriptomes and proteomes of flowering and non-flowering flower buds (FBs, anther and ovary differentiation completed) with different CRs. The concentrations of ABA and GA3 in the FBs were also determined using HPLC. The results indicate that chilling induced an upregulation of ABA levels and significant downregulation of *SHORT VEGETATIVE PHASE* (*SVP*) and *FLOWERING LOCUS T* (*FT*) homologs at the transcript level in FBs when the accumulated CR reached 570 CU (IB570) compared to FBs in November (FB.Nov, CK) and nF16 (non-flowering FBs after treatment at 16 °C for −300 CU), which suggested that dormancy breaking of FBs could be regulated by the ABA-mediated SVP-FT module. Overexpression in *Arabidopsis* was used to confirm the function of candidate genes, and early flowering was induced in *35S*::*CpFT1* transgenic lines. Our data provide insight into the minimum CR (570 CU) needed for chilling-induced dormancy breaking and its underlying regulatory mechanism in *C. praecox*, which provides a new tool for the artificial regulation of flowering time and a rich gene resource for controlling chilling-induced blooming.

## Introduction

Bud dormancy is a protective strategy for perennials to suspend growth for survival from adverse environmental conditions, which leads to the temporal insensitivity of plants to growth-promoting signals and the cessation of meristem activities^1^. Only when dormancy is released can plants resume growth under favorable environmental conditions^1^.

Photoperiod and temperature are the two central environmental signals that have a crucial role in the seasonal cycling of growth and dormancy^2–4^. The photoperiod is known to govern the cessation of perennial growth^5^. In autumn, short days (SDs) induce growth cessation of the shoot apical meristem (SAM), followed by the formation of buds and induction of dormancy before winter^6–8^. In contrast to photoperiod-controlled entry into dormancy, temperature primarily controls the duration of dormancy, and prolonged cold exposure induces dormancy breaking and bud btereak^9–11^. Interestingly, there is another situation in which low temperature (LT) can also cause growth cessation and control dormancy induction, regardless of photoperiod conditions^12,13^.

Phytohormones are known to have a crucial role in regulating the bud dormancy cycle of perennials^14^, and the complex process is mainly subject to the antagonistic regulation of gibberellins (GAs) and abscisic acid (ABA)^4,15^. GAs act as downstream targets of SDs in dormancy regulation^16^ and work in promoting dormancy release and bud break at a high level^17,18^. In contrast to GAs, ABA exerts antagonistic effects on dormancy regulation. SDs induces the accumulation of ABA and the expression of ABA signaling components to trigger dormancy establishment. High levels of ABA have been shown to maintain dormancy^19^. In contrast, ABA contents decrease towards dormancy release^6,20–23^, accompanied by a gradual increase in the GA level^24^.

Although the molecular mechanism underlying dormancy regulation is complicated and remains to be further understood, several critical regulators involved in this process have been described so far. In particular, the importance of dormancy regulated by ABA and the components downstream or upstream of ABA/GA have been identified.

The *dormancy-associated MADS-box* (*DAM*), a gene orthologous to the floral repressor SVP of *Arabidopsis*^25^, has been identified in the nondormant *evergrowing* mutant of peach^26^. This gene belongs to the STMADS11 subfamily of MADS-box genes, and its counterpart *SVP* transcriptionally inhibits the expression of *FT* in *Arabidopsis*^27^. *DAMs* showed distinct expression patterns in response to different environmental signals. Both SD and short-term LT treatments induce the upregulation of *DAM*s^28,29^, while a prolonged chilling requirement (CR) causes the reduction of *DAM* expression^30^. *DAM* homologs have also been characterized in other perennial species and have an essential role in the control of bud dormancy^31–35^. Overexpression of the *DAM* gene conferred early growth cessation and bud set in Japanese apricot^36^ and delayed bud break in apple^37,38^. PpDAM1 of pear upregulated the expression of the ABA biosynthesis enzyme 9-*cis*-epoxy-carotenoid dioxygenase (*NCED*) by binding to the CArG motif in the promoter of *PpNCED3*, which has an essential role in the regulation of bud dormancy^39^. Recent studies have provided insights into the roles of the poplar *SVP-LIKE* (*SVL*) gene in dormancy regulation. This gene negatively regulates *FT1* to affect bud break^19^. It also regulates *NCED3* expression directly, resulting in a high accumulation of ABA towards bud dormancy. Low temperature reduces ABA accumulation and represses the expression of *SVL* to promote bud break^19^. The overexpression of *SVL* was reported to suppress the dormancy defects of the *abi1-1* mutant in hybrid aspen^19^. Moreover, ABA mediates SD-induced *SVL* expression during bud dormancy^16^. In response to SDs, *SVL* induces the expression of *CALLOSE SYNTHASE 1* (*CALS1*), which mediates plasmodesmata (PD) closure to promote dormancy^16^. ABA can also induce the expression of *CALS1* by suppressing the expression of *PICKLE* (*PKL*), where dormancy defects can be restored in *abi1-1* plants^16,20,40^. ABA accumulation also contributes to PD closure to prevent dormancy release by restricting the transport of FT^20^. In contrast, the GA pathway is a downstream target of *SVL* in temperature-controlled bud break^19^. In addition, GA-mediated callose hydrolyzation helps the passage of FT to promote dormancy release^17^. The variation in *FT* gene expression levels during dormancy and after dormancy release is consistent with the observation that decreased expression of *FT* is induced by SD^19,34,41^, while LT upregulates the transcription of *FT* in breaking dormancy^17,42^. By comparison, *PaFT* has been shown to display increased expression levels in response to LT/SD in London plane trees^43^. Moreover, *PaFTL*, another *FT* orthologous gene in London plane trees, showed low and variable expression levels during dormancy and under LT/SD conditions^44^.

The *FT* gene functions as an upstream regulator of the *AP1*/*FUL* genes. In the SAM, the transcription of floral meristem identity genes *AP1*/*FUL* was stimulated by the FT/FD complex to promote flowering^45,46^. Interestingly, three *AP1/FUL*-like genes were observed to work upstream of *FT* homologs in rice^47^. The expression levels of *AP1* were observed to increase before dormancy release in *Ziziphus jujuba*^48^. Growth cessation was induced by SD-induced downregulation of *Like-AP1* (*LAP1*), an ortholog of the floral meristem identity gene *APETALA1* (*AP1*) in hybrid aspen^49^. However, similar to *LAP1* decreased expression of *PlacFL2* (*FUL-*like gene, an ancestor of *AP1*) was observed to control growth cessation mediated by SD downstream of CO/FT^50^.

*C. praecox* (wintersweet) is a crucial deciduous shrub that originated in China and is well known for its fragrant flowers and high ornamental value^51^. Adequate bud dormancy, followed by adequate bud breaking, is critical for its flowering and ornamental value. Warm winters, presumably due to global warming, have severe adverse effects on bud dormancy (and bud breaking, flowering, and plant performance) of wintersweet. Similar adverse effects have been observed in many other important plants, particularly horticultural crops such as apple (*Malus domestica*)^30,35,37,52–55^, blueberry, peach (*Prunus persica*)^56^, Japanese pear (*Pyrus pyrifolia*)^57–59^, and *Prunus* spp.^60–62^ In this study, we used wintersweet as a model to gain a comprehensive understanding of the fundamental molecular mechanism of floral bud dormancy in plants.

## Materials and methods

### Plant materials and chilling treatment

Eighteen-year-old potted *C*. *praecox* ‘Concolor’ plants and 15-year-old ‘Concolor’ and ‘Grandiflorus’ plants were grown on the campus of Southwest University (106°43E, 29°83 N, Beibei District, Chongqing City, China). Plants were exposed to forcing conditions at 12, 14, or 16 °C with different chilling requirement (CR) under SD conditions (8/16 h of light/dark) and 8000 lux illumination intensity in climate chambers (RDN-1000D-4, China) for a predetermined period from 8 Nov 2018 (average air temperature below 14 °C for three consecutive days). Richardson chill units (CU) for *C*. *praecox* ‘Concolor’ flower buds (FBs) were calculated according to the “UTAH Model”^63,64^. When the CU reached −300, 0, 150, 300, 450, and 570 (i.e., 16 °C for 600 h; 14 °C for 600 h; 12 °C for 300, 600, 900, and 1140 h, respectively), the treated plants were transferred to 14 °C for phenotype analysis. The following FBs were harvested and stored at −80 °C for sequencing and/or quantitative real-time PCR (qRT-PCR) analysis: FBs collected in April, May, and November (FB.Apr, FB.May, FB.Nov); FBs exposed to chilling conditions at 12 °C for 150, 300, 450 CU (FB150, FB300, FB450) and 570 CU (IB570, FBs initiate blooming); and nF16 (non-flowering FBs collected after treatment at 16 °C for −300 CU). Wintersweet plants cultured in open fields were used for the analysis of the influence of CR on chilling-induced dormancy breaking. The temperature data from Beibei meteorological station 57511# (106°27E, 29°51N, Beibei District, Chongqing City, China) were provided by the Chongqing Meteorological Bureau.

### Construction of libraries and RNA-seq

**Fig. 1:**
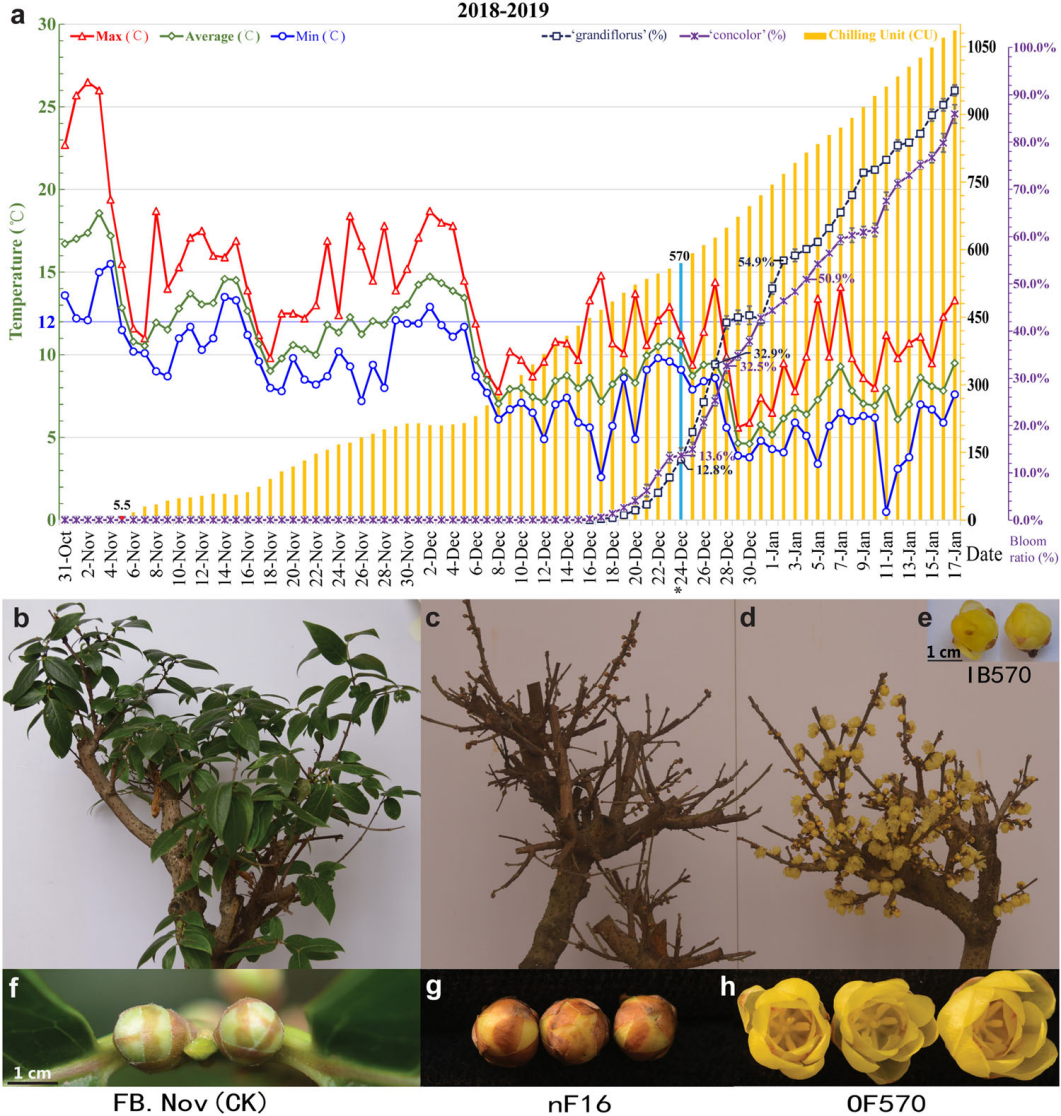
Chilling requirement determination under natural and artificial conditions. **a** Investigation under natural conditions in 2018–2019. The CR value was 5.5 on 5 Nov 2018. Blooming ratios of 12.8% and 13.6% occurred when CR accumulation reached 570 CU on 24 Dec 2018 in FBs from *C*. *praecox* ‘Grandiflorus’ and ‘Concolor’, respectively. **b**–**h** Artificial chilling-induced dormancy breaking and samples for omics analysis. **b** Potted *C. praecox* of eighteen-year-old in November as a control. **c** Plants subjected to −300 CU chilling requirement (CR) at 16 °C; FBs did not fully open and gradually dropped, showing the same phenotype as FBs subjected to 150, 300, and 450 CU at 12 °C (FB150, FB300, and FB450, photos not shown here). **d** Flowering plants subjected to 570 CU at 12 °C. **e** FBs initiated blooming after exposure to 570 CR (IB570). **f** FBs in Nov (FB.Nov). **g** Non-flowering FBs at 16 °C subjected to −300 CU (nF16). **h** Open flower stage after exposure to 570 CU (OF570). FB.Apr, FB.May, FB.Nov, FB150, FB300, FB450, nF16, and IB570 were used for RNA- and/or TMT-sequencing

A total of 24 independent RNA-Seq libraries from the FBs of eight groups, with three biological replicates for each group, were constructed and sequenced: FB.Apr, FB.May, FB.Nov (Fig. 1b, f), nF16 (Fig. 1c, g), FB150, FB300, FB450 (phenotypes just like nF16, photos not shown here), and IB570 (Fig. 1d, e). Briefly, a TRIzol reagent kit (Invitrogen, Carlsbad, CA, USA) was used to extract total RNA from each sample. Poly (A) mRNA was purified and fragmented into short lengths. The obtained fragments were used as templates, and first-strand cDNA synthesis was conducted using random hexamer primers, followed directly by second-strand cDNA synthesis. Next, the synthesized cDNA fragments were purified using a QIAquick PCR extraction kit (Qiagen, German); then, they were end-repaired, and poly(A) tails were added followed by ligation to Illumina sequencing adapters. These generated fragments were purified by agarose gel electrophoresis and then enriched by PCR amplification. Finally, the constructed cDNA libraries were sequenced on an Illumina HiSeq™ 4000 platform at Gene Denovo Biotechnology Co., Guangzhou, China. We deposited our sequencing data set in the NCBI Sequence Read Archive (SRA) under accession number PRJNA613935.

### Gene annotation, WGCNA, Venn diagrams, trends, and network analysis

Raw sequence reads were trimmed and checked for quality. The high-quality clean reads were subjected to de novo transcriptome assembly using Trinity^65^. Basic annotation of unigenes was performed based on the NCBI Nr, KOG, SWISS-PROT, and KEGG databases with E-values below 1e-5 as a threshold. Blast2GO software was employed to analyze the GO annotations of each unigene^66^. Subsequently, the functional classification of unigenes was conducted using WEGO software^67,68^. The number of reads per kilobase per million mapped reads (RPKM) was calculated and normalized to present the abundances of each unigene^66^. We used the edgeR package (version 3.12.1) to determine significant differentially expressed genes (DEGs) between two compared groups with a fold change threshold of 2 and a false discovery rate (FDR) threshold of 0.05. The calculated DEGs were then statistically enriched according to GO functions and KEGG pathways with an FDR value <0.05.

In addition, coexpressed modules and hub genes were detected by the Weighted Gene Co-expression Network Analysis (WGCNA, v1.47) package in R^69^ based on pairwise correlations between genes due to their similar expression profile. After filtering of the genes, the gene expression values from 70,458 genes were imported into WGCNA to construct co-expression modules using the automatic network construction function blockwiseModules (TOMType = ‘signed’, mergeCutHeight = 0.15, minModuleSize = 50, power = 12). Total connectivity and intramodular connectivity (function softConnectivity, connet = abs (cor(datExpr, use = “p”))^6, Alldegrees1 = intramodularConnectivity (connet, moduleColors)), kME (for modular membership, also known as eigengene-based connectivity, datKME = signedKME(datExpr, MEs, outputColumnName = “MM.”)) and kME-*P*-values were calculated for the 30,038 genes, which were clustered into 17 correlated modules. The other 57 genes were outliers (gray module) and are not presented. For those DEGs, we used hierarchically clustered heat maps to investigate their relative expression levels in different samples.

Venn diagrams were employed to calculate the DEG set in various comparisons. Trend analysis was performed using Short Time-series Expression Miner software (STEM)^70^. The networks were visualized using Cytoscape_v3.0.0.

### Protein extraction, protein digestion, and TMT labeling

The samples (FB.Apr, FB.May, FB.Nov, nF16, and IB570) were used for protein extraction. The total proteins from each sample were extracted using the cold acetone method^71^. The concentration of total extracted protein was measured using a BCA protein assay kit, and protein quality was confirmed by SDS-PAGE. Proteins were then tryptically digested quickly and entirely into peptides with sequence-grade modified trypsin (Promega, Madison, WI). After trypsin digestion, the peptide samples were centrifuged, drained by vacuum, and then reconstituted in 500 mM TEAB (triethylammonium bicarbonate). Each peptide mixture sample was labeled with various TMT 10-plex isobaric labeling tags (ThermoFisher Scientific, MA, USA) according to the manufacturer’s instructions by incubating at room temperature for 2 h. Subsequently, the labeled peptide mixture was redissolved and fractionated using high-pH separation. For each sample, twelve separated fractions were collected and dried for subsequent identification.

### LC–MS/MS, data, and functional analysis

Each collected peptide fraction was resuspended, separated, and analyzed on an Easy-nLC 1000 system (ThermoFisher Scientific, MA, USA) connected to an Orbitrap Fusion Tribrid mass spectrometer (ThermoFisher Scientific, MA, USA) equipped with an online nanoelectrospray ion source by Gene Denovo Biotechnology Co., Ltd., Mascot Distiller (version 2.6) was employed to extract tandem mass spectra, deconvolute the data, and deisotope charge states. The Mascot search engine (version 2.3.0.2, Matrix Science, London, UK) was used to identify and quantify the peptide/protein. In this study, the mascot database was set up based on the *C. praecox* reference transcriptome. The parameter settings for protein identification and quantitation were as described in a previous study^72^. We deposited our data set in iProX/ProteomeXchange under accession numbers IPX0001890001/PXD017101.

All identified proteins were annotated by searching against the GO, KOG, and KEGG databases. The significant enrichment of GO functions and KEGG pathways was determined using the hypergeometric test within differentially expressed proteins (fold change ≥ 1.2) with *P* ≤ 0.05. In addition, correlation analysis was performed for each comparison. To provide a valuable framework for a better understanding of the functional clustering of proteins, the protein–protein interaction network was constructed using the String program (http://www.stringdb.org/) with a confidence score threshold of 0.7. For some specific proteins, cluster heat maps were also used to reveal hierarchical clusters in the data matrices.

### Verification of RNA-seq data by qPCR and phylogenetic and functional analyses of *CpFT1* in *Arabidopsis*

To verify the reliability of RNA-seq data and determine the expression pattern of key DEGs related to chilling-induced dormancy breaking, qPCR was performed on a Bio-Rad CFX96 system using specific primers (Supplementary Table S1)^51^. The correlations between RNA-Seq and qRT-PCR were performed using the Spearman method, and the significance was analyzed with the Kruskal–Wallis test in R. The phylogenetic tree was constructed using neighbor-joining (NJ) bootstrap analysis (1000 replications) with the maximum composite likelihood model for DNA performed using MEGA6.0. The plant overexpression vector *35* *S*::*CpFT1* was transformed into wild-type *Arabidopsis thaliana* (Col-0) using the floral-dip method^73^. Finally, the transgenic plants, eight homozygous individuals from four different lines, were obtained by Kan-resistance screening for seeds, DNA identification of the T_1_ generation, and qRT-PCR analysis of T_2_ generation homozygotes of transgenic *Arabidopsis* using the primers listed in Supplementary Table S1. Three homozygous lines with different expression levels (high, medium, and low) were selected for follow-up analysis and phenotypic observations^73^. The significance of differences was analyzed by Student’s *t*-test.

### Quantification of ABA and GA by HPLC

To understand the endogenous hormone levels in chilling-induced dormancy, the FB samples, FB.Nov, FB150/300/570, IB570, LB570, and WP570, potted for artificial chilling experiments from *C*. *praecox* ‘Concolor’ were quantified by high-performance liquid chromatography-mass spectrometry (HPLC-MS) analysis of citrate-buffered acetone extracts in three biological replicates. The plant hormones (ABA and GA3) were analyzed qualitatively using HPLC and the relative retention time by comparison with reference standards. The ABA and GA3 isolates had retention times of 8.99 and 11.34 min, respectively.

## Results

### Chilling requirement for dormancy breaking

Based on paraffin sectioning, floral organ formation in wintersweet, including sepal, petal, stamen, and pistil primordia differentiation, was completed in April and May. Under natural conditions, wintersweet dormancy breaking occurred when CR reached 570 CU on 24 Dec 2018, and *C*. *praecox* ‘Grandiflorus’ and ‘Concolor’ had bloom ratios (BRs) of 12.8% and 13.6%, respectively. By 27 and 28 Dec 2018, the BR reached 32.9% and 32.5% under the accumulated CR of 627 CU and 649 CU, respectively, followed by 54.9% and 50.9% on 2 and 4 Jan 2019 under the accumulated CR of 768 CU and 815 CU, respectively (Fig. 1a).

For *C*. *praecox* ‘concolor’, from 18 Dec 2018 to 14 Jan 2019, the bloom ratio continued to increase from 1.3% to 75.1% under a cumulative CR from 485 to 1026 CU. To further clarify the CR for chilling-induced dormancy breaking, plants in November (FB.Nov, Fig. 1b) were treated at 12 °C or 16 °C with different CRs under the same light/dark period and light intensity conditions in the climate cabinet. After the CU reached −300, 150, 300, 450, and 570, the plants were transferred to 14 °C for phenotype observation. The results showed that only FBs under LT and SDs (8/16 h of light/dark at 12 °C) with a CR of 570 CU (IB570, Fig. 1e) could accomplish dormancy breaking and fully open (Fig. 1d, h). The FBs of nF16 (Fig. 1c, g) as well as FB150, FB300, and FB450 at 12 °C showed the same morphological phenotype as FB.Nov (CK), i.e., they could not expand and open normally, and the FBs gradually fell off.

### Sequencing, assembly, and annotation of the *C. praecox* reference transcriptome

To obtain a reference transcriptome from *C*. *praecox* FBs, RNA-seq libraries were constructed using RNA samples including five FBs under different CR conditions (nF16, FB150, FB300, FB450, and IB570) and three FBs collected in April, May, and November (FB.Apr, FB.May, and FB.Nov). A total of 100,553 contigs were generated using Trinity^65^, with total residues of 82,113,056 bp and an N50 of 1,252 bp. The average length of each transcript was 816 bp, the shortest sequence was 201 bp, and the longest was 16,864 bp. A total of 36,779 unigenes were annotated, of which 36,565 (36.4%), 24,758 (24.6%), 21,727 (21.6%), and 13,556 (13.5%) unigenes showed significant similarity to known genes in the NR, Swissprot, KOG, and KEGG databases, respectively. Approximately 27.8% (10,226) of unigenes could be assigned to a homolog in all four databases (Supplementary Fig. S1a), and the distributions of E-values in each database are shown in Supplementary Fig. S1b. A large number of unigenes in *C*. *praecox* showed close identities to the genes in other plant species. The numbers of homologous genes in the top 10-hit species are shown in Supplementary Fig. S1c. The highest number of *C*. *praecox* homologous genes (9537, 26.1%) was identified in a basal eudicot, *Nelumbo nucifera*, suggesting a closer phylogenetic relationship between *C*. *praecox* and *N. nucifera*^74^.

### Enrichment analysis of DEGs based on GO, KOG, and KEGG

Gene ontology (GO) enrichment analysis was carried out using a threshold value (*P* ≤ 0.05), and the primary biological functions of DEGs were classified into biological process (BP, 25,627; 48.19%), molecular function (MF, 16,250; 30.56%), and cellular components (CC, 11,302; 21.25%) (Supplementary Fig. S2a and Supplementary Table S2). Among the annotated DEGs of IB570/FB.Nov, the BP category contained the majority of GO annotations (1,515/3,335 up-/downregulated), followed by CC (868/1,843 up-/downregulated) and MF (710/1,514 up-/downregulated), in which developmental processes and rhythmic processes contained 50/120 and 1/3 up-/downregulated GO annotations, while in the case of IB570/nF16, the BP category contained 2009/1650 up-/downregulated annotations, in which the developmental process contained 68/54 up-/downregulated annotations (Supplementary Fig. S2b and Supplementary Table S3). The IB570/FB.Nov and IB570/nF16 clusters included 314 and 330 genes, respectively, and were enriched in GO categories related to floral development, organ development, and secondary metabolite production (Table 1 and Supplementary Table S4). IB570/FB.Nov or IB570/nF16 GO enrichment of the BP category contained 19 or 16 flower development (GO:0009908) annotations and 18 or 15 floral organ development (GO:0048437) annotations (Table 1 and Supplementary Table S4).

**Table 1:** Gene ontology (GO) categories enriched in IB570/FB.Nov and IB570/nF16

GO term	Ontology	Description	IB570/FB.Nov				IB570/nF16			
			GeneRatio	BgRatio	*P*-value	*p*.adjust	GeneRatio	BgRatio	*P*-value	*p*.adjust
*Floral development*										
GO:0009908	BP	Flower development	19	61	0.019	0.585	16	61	0.011	0.196
GO:0048569	BP	Postembryonic organ development	/	/	/	/	10	38	0.038	0.450
GO:0048437	BP	Floral organ development	18	50	0.004	0.341	15	50	0.003	0.078
GO:0010208	BP	Pollen wall assembly	6	17	0.093	0.935	2	17	0.726	1.000
GO:0048444	BP	Floral organ morphogenesis	8	19	0.019	0.585	8	19	0.003	0.071
GO:0048449	BP	Floral organ formation	8	19	0.019	0.585	8	19	0.003	0.071
GO:0048563	BP	Postembryonic organ morphogenesis	8	19	0.019	0.585	8	19	0.003	0.071
GO:0009555	BP	Pollen development	10	29	0.040	0.622	3	29	0.809	1.000
*Organ development*										
GO:0007275	BP	Multicellular organism development	/	/	/	/	80	491	0.123	0.823
GO:0048646	BP	Anatomical structure formation involved in morphogenesis	15	45	0.019	0.585	12	45	0.022	0.335
GO:0048856	BP	Anatomical structure development	123	547	0.034	0.616	92	547	0.056	0.529
GO:0009808	BP	Lignin metabolic process	/	/	/	/	1	15	0.903	1.000
GO:0042546	BP	Cell wall biogenesis	/	/	/	/	10	35	0.022	0.335
GO:0010927	BP	Cellular component assembly involved in morphogenesis	/	/	/	/	2	18	0.755	1.000
GO:0071669	BP	Plant-type cell wall organization or biogenesis	/	/	/	/	8	38	0.171	0.865
GO:0045229	BP	External encapsulating structure organization	65	222	0.000	0.125	47	222	0.004	0.078
*Secondary metabolite production*										
GO:0009698	BP	Phenylpropanoid metabolic process	20	54	0.002	0.216	5	54	0.905	1.000
GO:0009699	BP	Phenylpropanoid biosynthetic process	14	32	0.001	0.183	3	32	0.859	1.000

In a further analysis, the GO annotations were subjected to a search against the Clusters of Orthologous Group for eukaryotic complete genomes (KOG) database for functional classification and prediction. Based on sequence homology, 35,354 unique sequences were assigned a KOG functional classification. These sequences were classified into 25 KOG categories, denoting involvement in cellular processes, signal transduction, metabolism, and other processes (Supplementary Fig. S2c and Supplementary Table S5). To further determine the involvement of metabolic pathways in the flower opening process, we predicted the 132 KEGG pathways represented by all the assembled unigenes. The map with the highest unigene representation was the metabolic pathway (ko01100) with 2953 (39.08%) unigenes. Starch and sucrose metabolism (ko00500), plant hormone signal transduction (ko04075), and circadian rhythm (ko04712) contained 373 (4.94%), 326 (4.31%), and 72 (0.95%) unigenes ranked 7th, 10th, and 60th, respectively, of the total 132 pathways, including *PHYC*, *PRR73*, *GI*, *FT1*, and *FT2* (Supplementary Table S6). The top 20 KEGG pathway enrichments in IB570/FB.Nov are shown in Supplementary Fig. S2d (Supplementary Table S7).

### Correlation of differentially expressed genes (DEGs) and WGCNA

Compared with FB.Nov, nF16, and FB150/300/FB450, there were 9,884/11,817, 10,987/6,815, 20,011/36,130, 20,585/36,602, and 19,623/38,169 up-/downregulated genes in IB570, respectively (Supplementary Fig. S1d). There were 167/158 upregulated and downregulated proteins in IB570/FB.Nov and 25/50 in IB570/nF16 (Supplementary Fig. S1e).

**Fig. 2:**
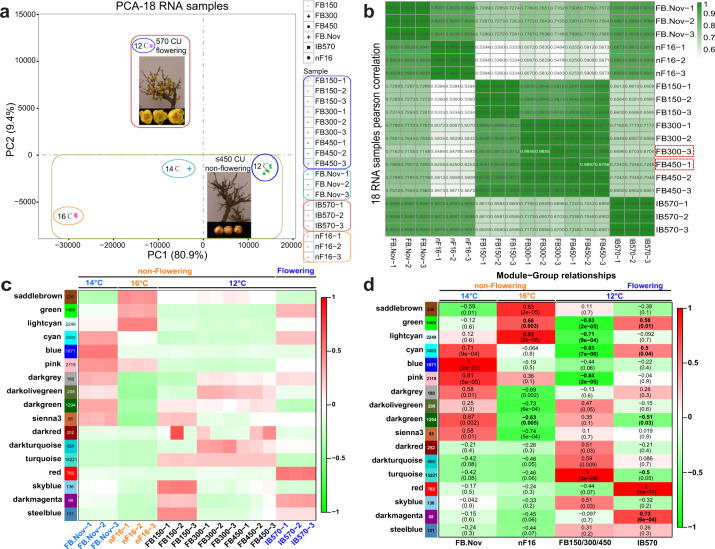
PCA, Pearson correlation, and WGCNA. **a** PCA plot of the 18 RNA-seq libraries. **b** Pearson correlation of the 18 RNA-seq samples. Red, dashed rectangles are placed around outlying libraries. **c** Module-tissue association. **d** Module-group relationship. Each row corresponds to a module. The major tree branches constitute 17 modules labeled by different colors. Each row corresponds to a module. The number of genes in each module is indicated on the left. The columns correspond to FB.Nov, nF16, FB150/300/450, and IB570

Principal component analysis (PCA) within the R package DESeq2^75^ and the average linkage cluster tree analysis within the WGCNA R package^69,76^ were carried out to screen for outlying libraries. The PCA revealed that the libraries were segregated horizontally (PC1) based on FB.Nov, nF16, and FB150/300/450 samples. Vertical segregation (PC2) clearly differentiated flowering (IB570) and non-flowering samples under different CR values, including FB.Nov, nF16, and FB150/300/450 (Fig. 2a). The Pearson correlation among three biological replicates of one sample was 0.98–1, while that among FB150, FB300, and FB450 was 0.87–0.99 and that between FB150/300/450 and IB570 was 0.66–0.72 (Fig. 2b). All the biological replicates had a strong correlation higher than 0.98 among three replicates of one sample, including libraries FB300-3 and FB450-1 (Fig. 2b).

Chilling-induced dormancy breaking**-**related network modules and gene expression patterns were identified by WGCNA and STEM. The linkage cluster tree revealed that libraries FB300-3 and FB450-1 did not group with their corresponding biological replicates (Supplementary Fig. S3a). Non-flowering samples under different CR values (FB150, FB300, and FB450) at 12 °C were clustered in the same group (Supplementary Fig. S3a). They are shown in the dendrogram, in which each leaf on the branch is one gene and each branch on the tree constitutes a module (Supplementary Fig. S3b and Supplementary Table S8).

**Fig. 3:**
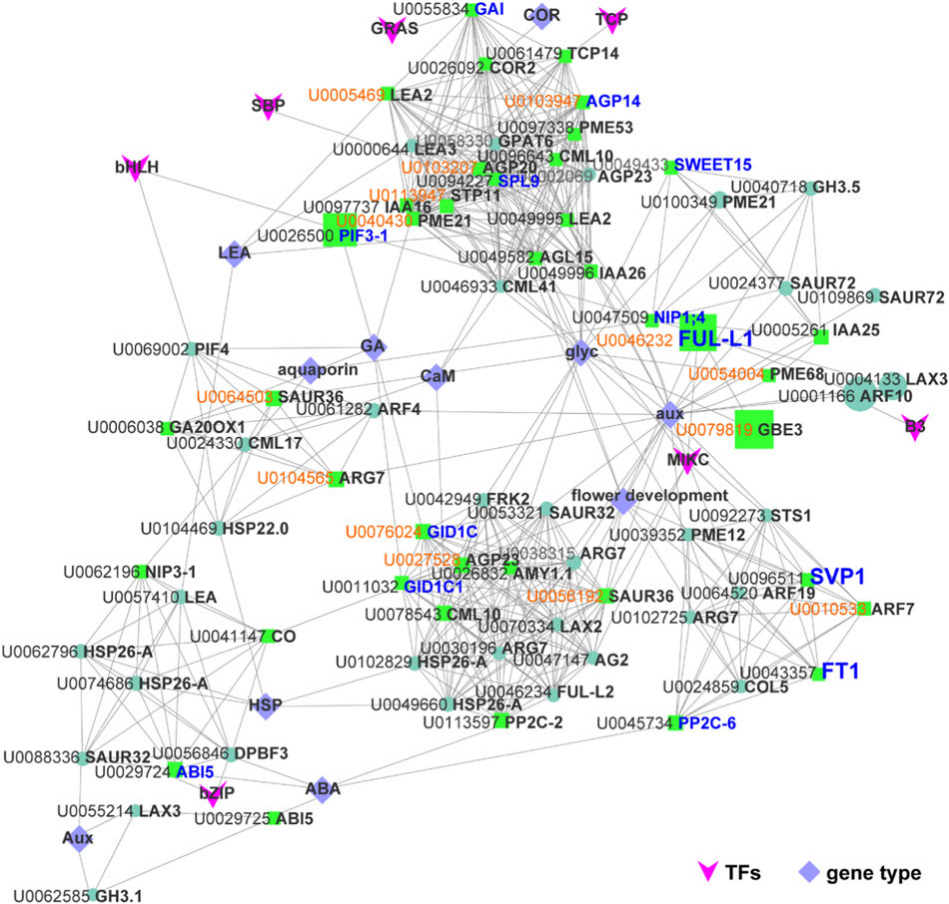
The co-expression networks of 76 DEGs based on Cytoscape. A total of 161 genes out of 701 selected genes (including 168 hub genes, containing 136 TFs, among which 45 TFs were hub TFs) were scanned for further gene network analysis by Cytoscape (Supplementary Table S9). Seventy-six DEGs out of the 161 genes belonged to 11 modules, the top five of which were red, light cyan, dark green, pink, and blue, with 19, 16, 9, 8, and 7 DEGs, respectively. There were 14 hub genes, and those with the top 5 highest total and intramodular connectivity were *GBE3*, *FUL-L1*, *ARF10*, *PIF3*, and *LAX3* (Supplementary Table S9). Each colored circle or square (node) represents one gene. A larger node size indicates greater connectivity within the network. Unigene (U) ID in orange represents the hub gene (kME > 0.95, and *P* < 10E−8)

**Fig. 4:**
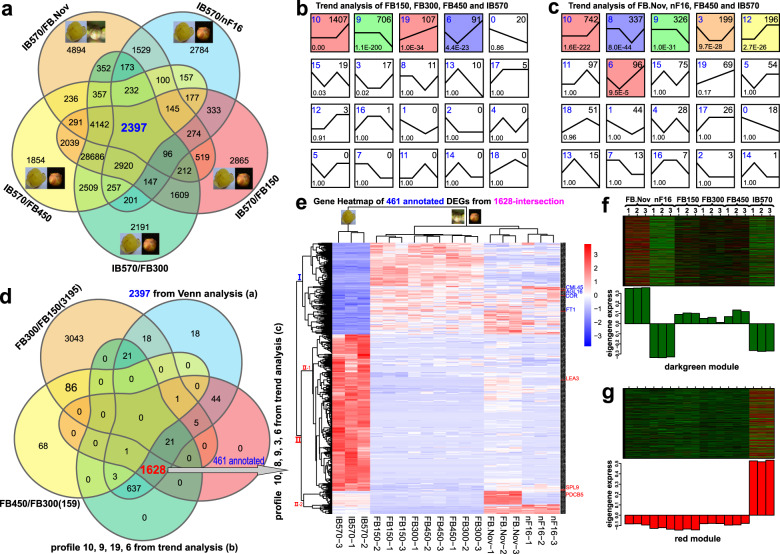
Venn diagram, trend, expression, and heat map analysis. **a** Venn diagram of DEGs from IB570/FB.Nov, IB570/nF16, IB570/FB150, IB570/FB300, and IB570/FB450. **b**, **c** Trend analysis of FB150, FB300, FB450 and IB570 (**b**) or FB.Nov, nF16, FB450, and IB570 (**c**) from the 2397 intersection in **a**. **d** Venn diagram of DEGs from FB300/FB150 and FB450/FB300, the 2397 intersection in **a**, 2311 DEGs from profiles 10, 9, 19, and 6 in **b**, and 1700 DEGs from profiles 10, 8, 9, 3, and 6 in the trend analysis in **c**. **e** Heat map of normalized RPKM scaled by the *Z*-score method for 461 annotated DEGs out of 1628 intersections in **d**, including *CYP707A1*, *FT1*, and *SPL9*. **f–g** Eigengene expression pattern of dark green (**f**) and red (**g**) modules

The 17 module eigengenes for the 17 distinct modules were each correlated with the CR phenotype due to the eigengenes’ CR phenotype-specific expression profiles (Fig. 2c, d). The CR combined with different temperature treatments and/or distinct phenotype networks incorporated 17 clusters (labeled by different colors) of coexpressed genes from a total of 30,038 unigenes based on WGCNA (Fig. 2d). A total of 7,042 hub genes were scanned from the module with kME > 0.95 and *P* < 10e−8. The module eigengene can be considered representative of the module’s gene expression profile for the first principal component of a given module. Notably, based on module-group relationship analysis (Fig. 3d and Supplementary Table S8), 7 out of 17 co-expression modules comprised genes that were highly expressed between a flowering tissue type (IB570) under 570 CU and non-flowering ones (FB150/300/450 or nF16), such as green, light cyan, cyan, dark green, turquoise, dark magenta (*r* ≥ 0.5, *P* ≤ 0.05), and especially red (*r* = 1, *P* = 1e−17). Among these modules, the correlation coefficients of turquoise-green, turquoise-cyan, green-dark green, and turquoise-light cyan ranged from −0.85 to −0.72 *(p* = 7e−6 ~ 8e−4) and that of dark green-light cyan was −0.51 (*p* = 0.03), while in the case of green-cyan, cyan-red, green-red, red-dark magenta, and green-light cyan, the correlation coefficients were between 0.5 and 0.76 (*p* = 0.04 ~ 3e−04; Supplementary Fig. S3c, correlation coefficients and *P*-values are marked in blue; Supplementary Table S8, the correlation coefficient is represented in a different color). Heat maps of the modules showing the relative normalized RPKM were generated to visualize the gene expression patterns over time, in which the largest of the modules was turquoise, containing 15,221 (50.7%) genes, followed by cyan, light cyan, pink, blue, green, dark green, and red (Figs. 2c, d, 4f–g and Supplementary Fig. S3d–i).

WGCNA can also be employed to construct gene networks in which each node represents a gene and the connecting lines (edges) between genes represent co-expression correlations. Hub genes are those that show the most connections in the network (kME > 0.95, and *P* < 10E−8). The *FT* homolog *CpFT1* (Unigene0043357) was correlated with *CpSVP1* and *CpPP2C-6* (Unigene0096511, *R* = 0.59 and Unigene0045734, *R* = 0.7; Fig. 3; Supplementary Table S9), which were found in gene sets 2397 and 1628 (Fig. 4a, d and Supplementary Tables S9, S11).

### Venn diagrams, trends, and heat map analysis

To filter preferential chilling-induced dormancy breaking-related DEGs, the 2,397—intersection of IB570/FB.Nov, IB570/nF16, IB570/FB150, IB570/FB300, and IB570/FB450 was identified using a Venn diagram (Fig. 4a). To reflect the major trends and the key transitional states among different CR tissues (Fig. 4b) and diverse temperature-treated samples (Fig. 4c), 2,397 selected DEGs were assigned to different profiles by STEM, and four and six profiles (profiles 10, 9, 19, and 6 from the trend analysis of FB150, FB300, FB450, and IB570; profiles 10, 8, 9, 3, 12, and 6 from FB. Nov, nF16, FB450, and IB570) were significantly enriched (Fig. 4b, c). After excluding the union of FB300/FB150 and FB450/FB300, together with 2,397 intersections (Fig. 4a), 2,311 DEGs (profiles 10, 9, 19, and 6 in Fig. 4b), and 1,700 DEGs (profiles 10, 8, 9, 3, and 6 in Fig. 4c) for another Venn analysis, 1,628 intersections were selected for further analysis (Fig. 4d). A heat map depicting the expression of 461 annotated DEGs (Supplementary Table S10) was constructed. Beyond the flowering samples of IB570, non-flowering samples of FB150/FB300/FB450 and nF16, and FB.Nov (CK) clustered together (Fig. 4e). Downregulation or upregulation of IB570 compared with FB.Nov, nF16, FB150, FB300, and FB450 was observed in clusters I (such as *CYP707A1*, *FT1*) and II-1 (*PDCB5*), which showed a positive correlation with eigengene expression in the dark green or red module from WGCNA, such as *CBL7* and KEGG significantly enriched *FT1* in the circadian rhythm-plant pathway (Supplementary Fig. S4), or Unigene0000644 *LEA3* and Unigene0094227 *SPL9*. The *COR* from the red module showed a negative correlation with its eigengene expression (Fig. 4e–g).

### Proteome/transcriptome association analysis

**Fig. 5:**
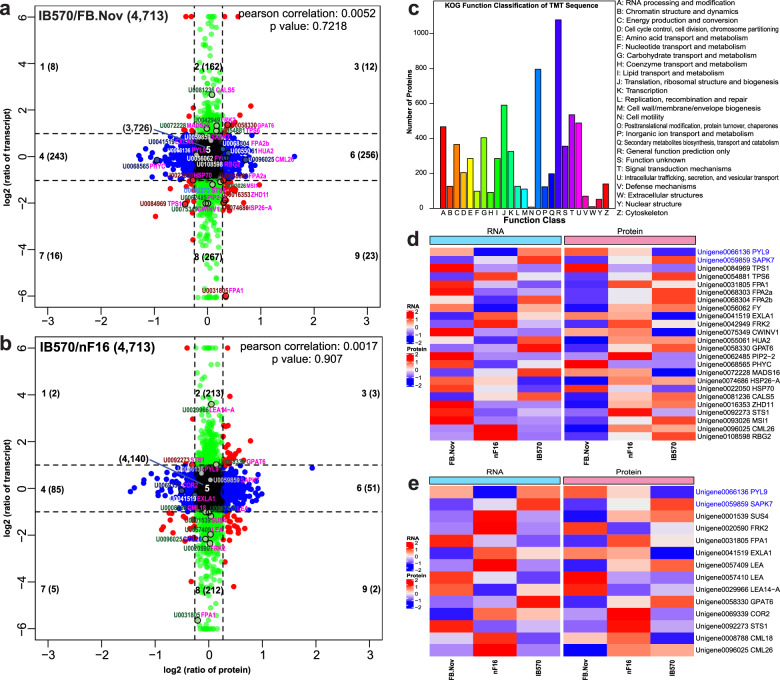
Nine-quadrant associate analysis, KOG functional classification of the TMT protein sequence, and heat map analysis of RNA and proteins. **a**, **b** Scatter plot of 9-quadrant associate analyses of mRNA and proteins from log_2_ FC (IB570/FB.Nov) and (IB570/nF16). Number 1–9, quadrant NO. The number of points in each quadrant is shown in parentheses. Unigene ID highlighted 24 or 14 transcripts and/or proteins. **c** KOG functional classification of differentially expressed proteins (fold change ≥ 1.2). **d**, **e** Heat map analysis of 24 (**d**) or 14 (**e**) normalized RPKM values scaled by the *Z*-score method from RNA and proteins in FB.Nov, nF16, and IB570

To identify the key genes for dormancy breaking and to complement the transcriptome results, we performed a comparative proteome survey by using TMT. To identify the scenarios that were in action, we compared the log_2_ Fold Change between IB570 and FB.Nov or nF16 samples in the transcriptome and proteome data sets from IB570, FB.Nov, and nF16. In group IB570/FB.Nov, 8, 162, 12, 243, 3,726, 256, 16, 267, and 23 transcripts/proteins were annotated in quadrants 1–9, compared with 2, 213, 3, 85, 4,040, 51, 5, 212, and 2 in the case of IB570/nF16. Of the 4,713 proteins with both mRNA and protein values, 20.9% (987 proteins) or 14.3% (673 proteins) showed at least 2- or 1.2-fold differences in mRNA and/or protein levels according to the log_2_ (ratio) from IB570/FB.Nov or IB570/nF16. A scatter plot of the nine-quadrant association analysis was then subdivided into sectors, and each was assessed for gene ontology terms that showed a statistically significant increase in protein levels only for FB.Nov or nF16 (quadrant 4), in mRNA only for IB570 (quadrant 2), in protein only for IB570 (quadrant 6), and in both protein and mRNA for IB570 (quadrant 3) (Fig. 5a, b). Interestingly, the majority (25.9% or 256 proteins; quadrant 6) showed significant fold changes (IB570/FB.Nov) only in protein abundance and not in mRNA, implying that the increase in most proteins in IB570 was independent of synthesis (that is, transcription and/or translation) (Fig. 5a), whereas in the case of IB570/nF16, the majority (1.8% or 85 proteins; quadrant 4) suggested downregulation of most proteins in IB570 (Fig. 5b).

A total of 7,344 TMT proteins were classified into 25 KOG categories, denoting involvement in RNA processing and modification, cell wall/membrane/envelope biogenesis, and other processes (Fig. 5c and Supplementary Table S11). Labels in the nine-quadrant association analysis diagram and heat map analysis were obtained using 24 and 14 selected unigenes and the predicted proteins from IB570/FB.Nov or IB570/nF16. Protein expression was upregulated in IB570 cells compared with FB.Nov, such as Unigene0096025 CML26, Unigene0068304 FPA, and Unigene0056062 FY from quadrant 6, Unigene0068303/Unigene0031805 FPA from quadrant 9, which was downregulated in Unigene0068565 PHYC, Unigene41519 EXLA1 from quadrant 4, and Unigene0084969 TPS1 and Unigene0022050 HSP70 from quadrant 7 (Fig. 5a, d). In group IB570/nF16, protein expression was downregulated in Unigene0069339 COR2 from quadrant 4 (Fig. 5b, e). At the mRNA level, Unigene0059859 SAPK7 expression in IB570 was significantly higher than that in FB.Nov and nF16 by ca. 1.61- and 1.28-fold, respectively (Fig. 5).

### Verification and phylogenetic analysis of DEGs during dormancy breaking

**Fig. 6:**
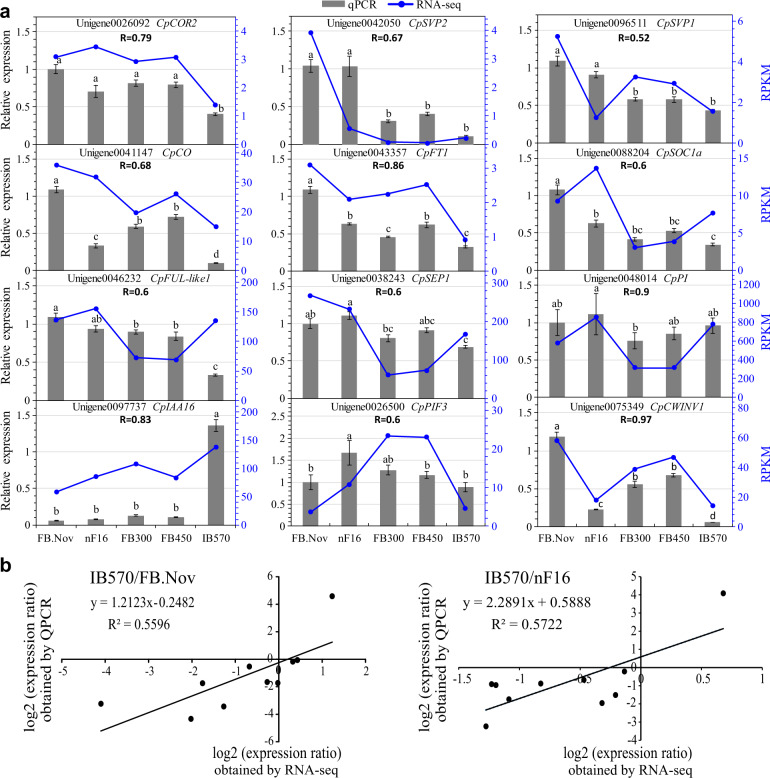
Consistency and linear regression analysis of the transcriptome by qRT-PCR. **a** Verification of the consistency of the RNA-Seq data using qRT-PCR in FB.Nov, nF16, FB300, FB450, and IB570. The different letters show significant differences at *P* = 0.05 based on a *t*-test. **b** Linear regression analysis of IB570/FB.Nov and IB570/nF16

The transcript abundance of 12 DEGs was analyzed to verify the reliability of the RNA-Seq data using qRT-PCR (Fig. 6a). Linear regression analysis showed an overall correlation coefficient (*R*) = 0.75 and 0.76 for IB570/FB.Nov and IB570/nF16, respectively, which indicates a good correlation between the qRT-PCR results and transcription profile, with corresponding Spearman correlations of *R* = 0.52–0.97 (Fig. 6a, b). The relative expression level in IB570 was significantly downregulated compared with that in FB.Nov and nF16, respectively, such as *COR2*, *SVP1*, *SVP2*, *CO*, *FT1, SOC1a*, *FUL-like1*, *SEP1*, and *CWINV1*; among them, *COR2*, *CO*, *FUL-L1*, and *CWINV1* were also downregulated in IB570 compared with FB300 and FB450, while significant upregulation of *IAA16* was detected in IB570 compared to other samples (Fig. 6a).

Phylogenetic analysis of flower development-related DEGs showed that Unigene0096511 *CpSVP1* and Unigene0042050 *CpSVP2* clustered closer to *SVP* in *Arabidopsis* and *PtSVL* in *Populus* than to Japanese apricot, peach, or pear *DAM*s, which comprise a larger subfamily of MADS-box genes, *SVP*/*AGL24*; Unigene0025911 *CpAGL6* and Unigene0046232 *CpFUL*-*L1* formed a clade with *AGL6* and *FUL* from the *AP1*/*SEP*/*AGL6* superclade of MADS-box genes (Supplementary Fig. S5).

### Concentration of ABA and GA and expression analysis

**Fig. 7:**
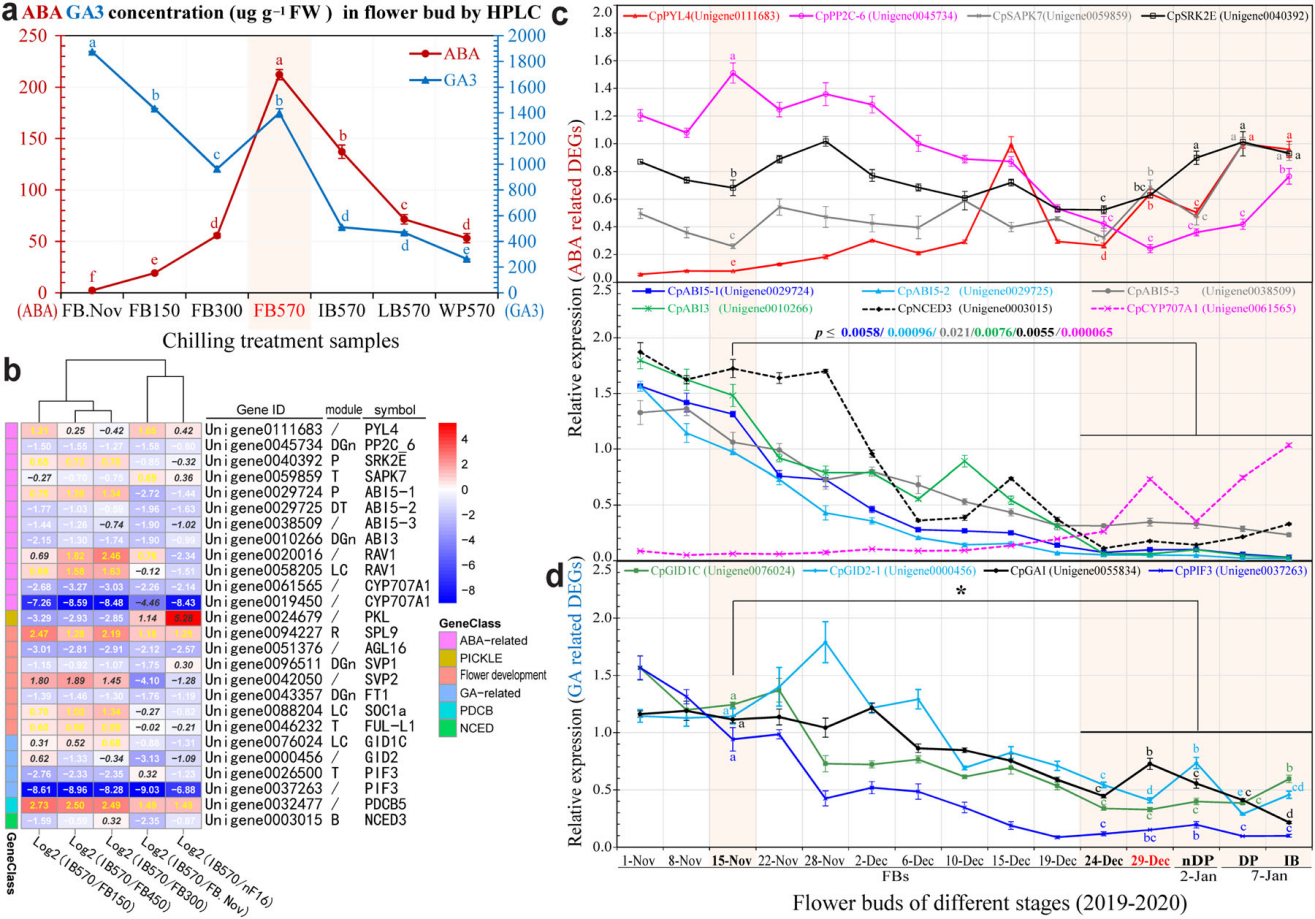
Concentration of ABA and GA determined by HPLC and heat map analysis of 26 DEGs. **a** Concentration of plant hormones (ABA and GA3) in samples under different artificial chilling treatments at different stages. **b** Heat map of 26 candidate genes based on the log_2_ (fold change) of RPKM scaled by the *Z*-score method. B, blue; DGn, dark green; DT, dark turquoise; LC, light cyan; P, pink; R, red; T, turquoise. Black bold italics, yellow bold/white bold numbers represent no significant difference, significant up-/downregulation at a fold change ≥ 1.5 (FDR ≤ 0.05). **c**, **d** Relative expression of ABA- and GA-related DEGs in FBs under natural conditions in 2019–2020. Different letters indicate significant differences (*P* < 0.05) in FBs on 15-Nov, 24-Dec, 29-Dec (567 CU), 2-Jan (nDP stage, 608CU), and 7-Jan (DP and IB stages) based on a *t*-test

The concentration of ABA in FBs increased significantly from FB.Nov to FB150 and FB300 reached a peak in FB570, then experienced a sustained decline from IB570 to LB570 and WP570. A significant decrease in the concentration of GA3 was detected from FB.Nov to FB150 and FB300, followed by an increase to FB570, and then a decreasing trend, similar to that of ABA, from IB570 to LB570 and WP570 (Fig. 7a). Through the analysis of the RNA-seq data and qPCR, we found that hormone-related genes changed greatly in the process of chilling-induced dormancy breaking, especially the genes related to ABA and GA biosynthesis and signal transduction pathways (Figs. 7b and Supplementary Fig. S6).

The following was also observed: significant upregulation of the abscisic acid receptor Unigene0111683 *PYL4* in IB570/FB.Nov and IB570/FB150, followed by the downregulation of protein phosphatase, Unigene0045734 *PP2C-6*, in IB570 compared with that in FB.Nov, nF16, and FB150/300/450, followed by the upregulation of serine/threonine-protein kinase SnRK2, Unigene0040392 *SRK2E* in IB570/FB150, IB570/FB300, and IB570/FB450 and Unigene0059859 *SAPK7* in IB570/FB.Nov (Fig. 7b). Significantly enriched *abscisic acid-insensitive 5* (*ABI5*, Unigene0029725, and Unigene0038509) and Unigene0010266 *ABI3* showed downregulation in pairwise comparisons, except for Unigene0038509 *ABI5–3* in IB570/FB300 and IB570/nF16, with no significant difference (Fig. 7b and Supplementary Fig. S6). Unigene0061565/Unigene0019450 *CYP707A1* was downregulated in five pairwise comparisons, except for Unigene0019450 in IB570/FB.Nov. The relative expression of *CpPP2C-6* (Unigene0045734), *CpABI3* (Unigene0010266), and *CpABI5-1/2/3* (Unigene0029724, Unigene0029725, Unigene0038509) in FBs on 29 Dec 2019 (567 CU, 1.42% BR) was *ca*. 6.3-, 25.7-fold, and 13.1-/19.4-/3*-*fold lower, respectively, than that on 15 November (0 CU). The expression of Unigene0061565 *CYP707A1* in nDP (nearly to the displayed petal stage, 2-Jan) was significantly reduced by ~2.1-fold compared to that in FBs on 29-Dec (Fig. 7c).

Two gibberellin receptor GIDs (Unigene0076024 *GID1C*, Unigene0000456 *GID2-1*) and transcription factor Unigene0026500/Unigene0037263 *PIF3* were downregulated in IB570 compared with FB.Nov and/or nF16. Downregulation of Unigene0026500/Unigene0037263 *PIF3* was detected in IB570 cells compared to FB150/300/450 cells (Fig. 7b). The expression of Unigene0076024/Unigene000456 *GID1C*/*GID2-1* and Unigene0037263 *PIF3* in FBs on 29 Dec was *ca*. 3.8-/2.8-fold and 6.3-fold lower, respectively, than that on 15 Nov (Fig. 7d and Supplementary Fig. S6).

**Fig. 8:**
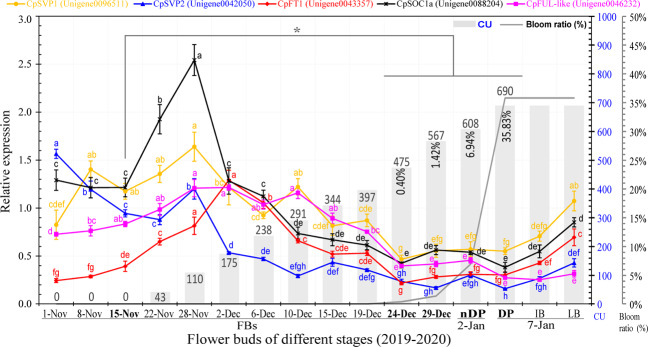
Flowering characteristics and relative expression in FBs under natural conditions in 2019–2020. Small letters show significant differences (*P* = 0.05 based on a *t*-test). nDP, nearly to the displayed petal stage (DP) before petal enlargement

The expression of *CpSVP1*, *CpSVP2, CpFT1, CpSOC1a*, and *CpFUL-L1* in FBs on 24-Dec (475 CU, BR of 0.4%), 29-Dec 2019 (567 CU, BR of 1.42%), 2-Jan (nDP stage, 608CU, BR of 6.94%) and 7-Jan 2020 (DP, displayed petal stage, 690CU, BR of 35.83%) showed significant downregulation compared to that in FBs on 15-Dec-2019 (Fig. 8).

### Functional analysis of *CpFT1*

**Fig. 9:**
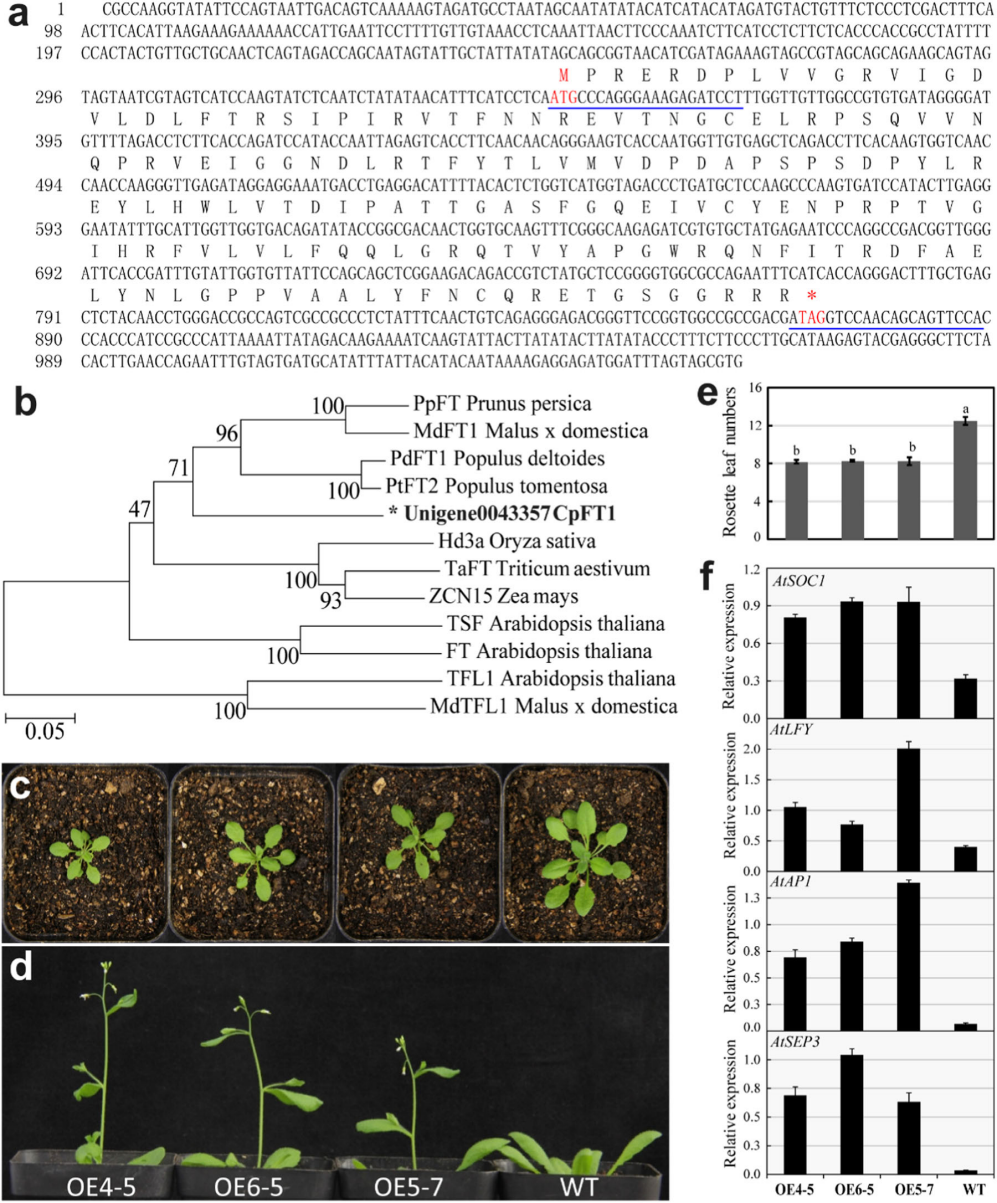
Phylogenetic and functional analyses of *CpFT1*. **a** The DNA and protein sequence of CpFT1. **b** Phylogenetic tree based on NJ bootstrap analysis. **c**, **d** Phenotype of the *35S*::*CpFT1*/Col-0 T2 generation. **e** Numbers of rosette leaves of transgenic lines compared with WT. **f** Relative expression analysis in ectopic expression lines of *CpFT1*. The blue underline shows the forward and reverse primers for the *35S*::*CpFT1* vector construction

*CpFT1* (accession number: MT565392) is 1,060 bp in length and has an open reading frame (ORF) of 525 bp, which encodes a predicted polypeptide of 174 aa with a 5′/3′-UTR of 346 and 189 bp (Fig. 9a). In the phylogenetic tree*, CpFT1* clustered close to Hd3a in *Oryza*, TaFT in wheat, and ZCN15 in maize (Fig. 9b). The ectopic expression results indicated that the time of bolting, the first flower opening, and the first fruit emerging in the *35S*::*CpFT1* transgenic lines (OE4–5, OE6–5, OE5–7) were significantly earlier than those in the wild-type (WT; Fig. 9c, d). The number of rosette leaves of transgenic lines (average 8.22) was lower than that of the WT (average 12.47) (Fig. 9e). The results of qRT-PCR for different *35S*::*CpFT1* lines and WT showed that the heterologous expression of *CpFT1* in *Arabidopsis* upregulated the expression levels of *SOC1*, *LFY*, *AP1*, and *SEP3*, which resulted in early flowering in *Arabidopsis* (Fig. 9f). Therefore, it is inferred that the overexpression of *CpFT1* can promote flowering in *Arabidopsis*.

## Discussion

Prolonged exposure to cold temperatures, also known as chilling, regulates dormancy release, including bud break or bud flush^77^. In *Prunus mume*, floral meristem (FM) differentiation is completed in summer, while blooming occurs until the next early spring^56^. In the case of wintersweet, paraffin sections showed that FM formed from March to early April, followed by tepal primordium differentiation from the end of April to early May, followed by stamen and pistil primordium in mid- and late May. The development of anthers stagnated from June to August, resumed in September, and entered the dormant period from October (Supplementary Fig. S7). Under natural conditions, FB experienced cold induction from mid- to late November and opened fully in late December to January in Chongqing.

*P*. *mume* cultivars ‘Nanko’ and ‘Ellching’, from temperate Japan and subtropical Taiwan, require ~500 and 300 “chilling hours” (CHs), respectively, to break FB dormancy^78^. Peach cultivars with CRs ranging from fewer than 50 CU to more than 1000 CU have been developed and used for breeding and cultivation worldwide^56^. The results from our experiment showed that all the wintersweet plants in the field or potted plants underwent the CR for dormancy breaking, reaching 570 CU. The FBs expanded and fully opened, although temperature fluctuation may result in the CR value for chilling-induced dormancy breaking in wintersweet cultured in the field being slightly higher than that in the artificial climate chamber under LT (12 °C) and SDs conditions. Chilling below 7 °C is not the necessary condition for dormancy breaking in wintersweet. In Guangzhou City, Guangdong Province, the FBs of wintersweet cultured in the open field did not fully open (just like nF16), similar to the nonchilled peach FBs, which were almost undeveloped even at 20–23 °C for a few months. Early work was based on the sum of hours at a temperature <7.2 °C (chilling hours), which were added throughout the estimated period of dormancy in peach^79^. The “UTAH Model” proposes records on the number of “chill units”, establishing a different chilling contribution for different temperature ranges^63^, which is more suitable for chilling requirement analysis in chilling-induced dormancy breaking in wintersweet.

**Fig. 10:**
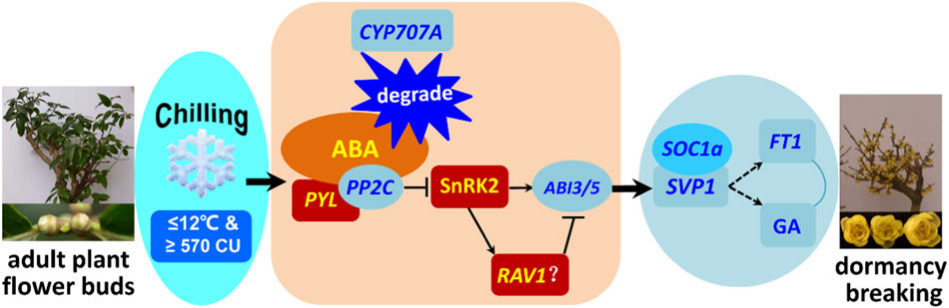
Pathway of chilling-induced dormancy breaking in *C. praecox*. See the “Discussion” section for details. Red or orange and gray-blue or sky-blue colors denote up- and downregulation, respectively. The dashed lines and arrows indicate potential repression and indirective promotion, respectively

Previously, a group of genes encoding MADS-box TFs called *DAM*s, phylogenetically related to *A. thaliana SVP* and *AGL24*, was recruited to regulate the dormancy cycle in perennial woody plants^80^. For example, a negative correlation between the *PpDAM5* and *PpDAM6* transcript levels of lateral vegetative buds and the bud burst ratios and flower organ expansion rate was found in the low- and high-chill field cultivars of *P. persica*^81^. After organ differentiation, a prolonged period of artificial chilling is necessary for the reduction of the *PpDAM5* and *PpDAM6* transcript levels in the FBs, which are recruited in flower organ enlargement^29,82^. Six *PmDAM* genes in *P. mume* showed downregulation following prolonged artificial chilling exposure. A certain CR may be necessary for the downregulation of *PmDAM4* to *PmDAM6* in high-chill ‘Nanko’^56^. The distinct changes in *PmDAM4* to *PmDAM6* expression may contribute to the different CRs for dormancy release in ‘Ellching’ and ‘Nanko’^36^. PpDAM proteins downregulated the expression of *PpFT2* during dormancy release in *P. pyrifolia* ‘Suli’^34^. In addition, *MdDAMs* showed seasonal mRNA fluctuation patterns and were downregulated by artificial cold exposure^30^. In hybrid aspen, *SVL* has been shown to participate in the regulation of both the entry and release of dormancy as mediators of temperature-controlled bud break^16,19,77^. Although high similarity was found among SVL, SVP, and DAM, the *SVL* clusters were closer to the *SVP* than to the *DAM* genes^19^. Phylogenetic analysis of *CpSVP1/2* in *C. praecox* showed the same results as poplar *SVL* (Fig. S5). The downregulation of *SVL* in buds of hybrid aspen by prolonged exposure to LT was similar to the downregulation of the other *DAM* genes associated with bud break^28,33,83^. *SVL* negatively regulates *FT1*, one of two poplar *FT* paralogs (*FT1* and *FT2*), which have divergent functionally^84^. *FT* has an important role in flower initiation and participates in the regulation of dormancy in perennial trees^8,85^. *FT1* is highly upregulated by chilling in dormant vegetative buds^84^. In our study, *CpFT1* was significantly enriched in the KEGG circadian rhythm-plant pathway and associated with chilling-induced dormancy breaking (Fig. 4d, e). However, it was unexpected that the expression trend of *CpFT1* was highly consistent with that of *CpSVP1*, other than the negative regulation of *FT* by *SVP* in other species. One possible explanation for this result is that there is no *FLC* in the *C. praecox* genome. The repression of *FT* expression by SVP may depend on the FLC that can interact with SVP^86^. The transcription profiling of the chilling requirement for bud break in apples revealed strong differential expression in *FLC-like* and *MADS AFFECTING FLOWERING* (*MAF*); the upregulation of *FLC-like* showed a remarkable induction towards dormancy release^55^. Recently, it was reported that PavDAM1/5 could interact with PavSOC1 in vivo and in vitro and coregulate flower development in sweet cherries (*Prunus avium*)^87^. In wintersweet, CpSVP1 may interact with CpSOC1a to positively regulate *CpFT1*, so the downregulation of *CpSVP1* and *CpSOC1a* results in the downregulation of *CpFT1* during dormancy breaking (Fig. 10).

Hormone homeostasis has an important role in bud dormancy^4^. Two core hormones, ABA and GA, antagonistically regulate bud dormancy status. In hybrid aspen, a prolonged chilling signal reduces ABA levels and triggers a reduction in *SVL* expression, resulting in the upregulation of GA biosynthesis and *FT1* expression, which promotes bud break^19^. In *P. mume*, the ABA/GA ratio was reported to steadily decline during the dormancy release process^60^. In the present investigation, however, the ABA content significantly increased in FB570 compared with FB.Nov under artificial treatment, while the content of GA3 decreased (Fig. 7a), implying that the ABA/GA3 ratio significantly increased during chilling-induced dormancy breaking. During the process of FB enlargement and blossoming, the content of GA3 in FBs was much higher than that of ABA, which is consistent with observations in tree peony^88,89^. In support of this, we found a significant downregulation of the ABA 8-hydrolase gene (*CYP707A1*) and upregulation of the ABA synthesis gene *NCED* in FB570 compared to FB.Nov and FB150-450. Moreover, significant changes in the expression of the genes related to the ABA signaling pathway were also detected between FB570 and FB.Nov. For instance, the genes encoding ABA receptors (*PYL4*) and *SnRK2* kinase were significantly upregulated, while the ABA coreceptor *PP2C* was evidently downregulated in FB570, which is consistent with their roles in the ABA signaling pathway and the regular responses to a high ABA content (Fig. 10). Upon ABA signaling, PYR/PYL receptors interact with PP2Cs, thus disrupting the PP2C-SnRK2 interactions, which release SnRK2 kinases from PP2C-mediated inhibition, allowing SnRK2s to phosphorylate and activate downstream transcription factors such as ABI/ABFs^90^. The downregulation of the ABA pathway could result in the downregulation of *SVL* expression upon exposure to prolonged chilling in hybrid aspen^19^. In wintersweet, however, the downregulation of *CpSVP1* was accompanied by an increased ABA content, which may be explained as follows: although the ABA content increased during CR accumulation in wintersweet, *ABI3* and *ABI5* homologs were significantly downregulated in the FBs during the process of CR accumulation (Fig. 10), which is consistent with the results in other species during chilling-induced dormancy breaking, where the ABA content usually decreased. Recently, it was reported that ABA-responsive PpyABF3 can directly bind to the ABRE element in the promoter of *PpyDAM3* and activate its expression to promote bud dormancy in Asian pear (*P. pyrifolia*)^91^. In addition, *SOC1* was identified to be a direct downstream target of ABF3/4 in *Arabidopsis*, and the induction of *SOC1* by ABA was hampered in *abf3 abf4* mutants^92^. In wintersweet, the expression patterns of *CpSVP1* and *CpSOC1a* were consistent with those of the *ABF* homologs (*CpABI3* and *CpABI5s*), suggesting that CpABI3 and CpABI5s transcription factors may positively regulate the expression of *CpSVP1* and *CpSOC1a* (Fig. 10).

In *C. praecox*, the reactivation of blossoming (dormancy breaking followed by enlargement and blooming) in the FBs was induced by LT combined with SD conditions when the CR reached 570 CU. The hormone accumulation patterns in the FBs during chilling-induced dormancy breaking in *C. praecox*, that is, up- and downregulation of ABA and GA, are opposite to the situation in other species such as *Populus* during bud break. This may have resulted from the fact that bud break in *Populus* is different from FB dormancy breaking in *C. praecox*, which undergoes two different biological processes, vegetative bud growth as well as FB enlargement and blooming.

*AP1*/*FUL*-like genes have been reported to work downstream of *FT*-like genes or to be direct targets in other species^46,93–95^. In winter wheat and barley, similar regulation strategies of flower arrest and promotion to *Arabidopsis* were found, but different policies were exploited, including at least four critical regulators for vernalization-induced flowering, such as *VRN1* coding for AP1/FUL-like, *VRN2* for CO-like, *VRN3* for FT*, and PPD1* for PRR7^96–99^. *VRN2* and/or *VEGETATIVE TO REPRODUCTIVE TRANSITION2* (*VRT2, SVP* orthologous gene) repressed *VRN1* expression in wheat^100,101^. *VRN1* was proven to work upstream of *VRN3* (*WFT*) by binding to the CArG-box in the promoter region^102^; *OsMADS14*, *OsMADS15*, and *OsMADS18* (*AP1/FUL*-like genes) can directly upregulate *Hd3a* and *RFT1* (*FT* homologs) in rice^47^. Before bud hibernation, the *FUL*-like ortholog *PlacFL2* from the basal eudicot *Platanus* declined, implying that it may control SD-mediated dormancy and growth cessation^50^. Similar to *LAP1*, a tree ortholog of *AP1* participated in SD-mediated seasonal growth cessation downstream of the CO/FT module in hybrid aspen^49^. In this study, *CpSVP1* participated in the reduction of the *SVP*-mediated promotion of *CpFT1* and subsequently gave rise to *CpFUL-L1*. In addition, *CpFUL-L1* could give rise to *FT* expression levels in *35S*::*CpFUL-L1*/Col-0 transgenic *Arabidopsis* lines with an early flowering phenotype compared to WT (unpublished data).

In summary, we propose that the extended cold exposure signal results in the upregulation of the ABA content in FBs under SD and LT of 12 °C with a CR of 570 CU. An increase in ABA may be a result of the upregulated NCED and downregulated *CYP707A1*. ABA as a signal was accepted by *PYL4*, and ABA-PYL complexes can inhibit *PP2C*, which suppresses *SnRK2* function through dephosphorylation. Phosphorylated *SnRK2* can activate *RAV1*, which then inhibits the expression of *ABI3* and *ABI5s*. SD and short-term LT treatments induced the upregulation of *SVP1*, while prolonged CR caused the reduction of *SVP1/2*, which combined with *SOC1a* to induce *FT1* expression and reduction of GA, subsequently inducing the breaking of dormancy in *C. praecox*.

## Supplementary information

Table S1

Table S2

Table S3

Table S4

Table S5

Table S6

Table S7

Table S8

Table S9

Table S10

Table S11

Supplementary Figures
