# Supplementary material for: Integrated transcriptome and proteome analysis provides insight into chilling-induced dormancy breaking in Chimonanthus praecox
Source: Hortic Res. 2020 Dec 1;7:198. doi: 10.1038/s41438-020-00421-x (PMC7704649; doi:10.1038/s41438-020-00421-x)
Supplement: Supplementary file 1 — Table S1 [file 41438_2020_421_MOESM1_ESM.docx]

**Supplementary material**

**Table S1. List of primer pairs used in this study.**

| **Primer Name** | **Primer Sequence (5’-3’)** | **Description** |
| --- | --- | --- |
| q*CpActin*-F | GTTATGGTTGGGATGGGACAGAAAG | qRT-PCR  qRT-PCR |
| q*CpActin*-R | GGGCTTCAGTAAGGAAACAGGA |  |
| q*CpTublin*-F | TAGTGACAAGACAGTAGGTGGAGGT |  |
| q*CpTublin*-R | GTAGGTTCCAGTCCTCACTTCATC |  |
| q*CpCOR2*-F | CACCGTCGTCTATACAGATGGC |  |
| q*CpCOR2*-R | TTTCCATCGGAGTTGGCAGG |  |
| q*CpSVP1*-F | ATTGACAACACCACCGCTCG |  |
| q*CpSVP1*-R | TTGCTCAACATGGTGCAGCG |  |
| q*CpSVP2*-F | AGTGTTGAACCAGCAGGTAGCAG |  |
| q*CpSVP2*-R | CACCCTTCATTTGCAAGCTACTG |  |
| q*CpCO*-F | ATTTCCACTGGCGAAGATGTC |  |
| q*CpCO*-R | TTCACTCAACAGATGCTCCCCT |  |
| q*CpFT1*-F | CCGTGTGATAGGGGATGTTTTAG |  |
| q*CpFT1*-R | GTTGACCACTTGTGAAGGTCTGAG |  |
| q*CpSOC1a*-F | AGACAATCCTATTAGAAGAAAATGC |  |
| q*CpSOC1a*-R | TTCCCTTAAGTTCATAGACTTCAAT |  |
| q*CpFUL-like 1*-F | CTTGACGCCTTGAGTCTCAGAG |  |
| q*CpFUL-like 1*-R | AGTGAGCTTGGAGAGCTATCGC |  |
| q*CpSEP1*-F | ACAGAAGGAGTAGCAATGGTGGT |  |
| q*CpSEP1*-R | GCTCGTCAAGCAGAAACTGAGTC |  |
| q*CpPI*-F | GATTACTGTAGTCCCTCATCCACG |  |
| q*CpPI*-R | CACGAACACCAGCAAGACCATT |  |
| qCpGID1C-F | TAAGGACAGAGTAGAGGAGAAGCG |  |
| qCpGID1C-R | GCAACGCAAAGCCAAACAAG |  |
| qCpGID2-1-F | GCTTTGGGAGATGGTCTGTACC |  |
| qCpGID2-1-R | ATCCACCAAGGGCAAGAACG |  |
| qCpPIF3-1-F-(26500) | TTGAGTGTAGCCTTGGGAGTGTC |  |
| qCpPIF3-1-R-(26500) | TGAACTTCGGTGGATCGGCT |  |
| qCpPIF3-2-F-(37263) | GGGTCAAGCACTGTTGTCCA |  |
| qCpPIF3-2-R-(37263) | TGGCTCCACTAAGACTGTTGCT |  |
| qCpGAI-F | GAGTCGGTGATGGTGGGCAA |  |
| qCpGAI-R | AGTGGAGAAATCGGGGTTGAG |  |
| qCpPP2C-6-F | GCAGAATGGGATCAAGGAGC |  |
| qCpPP2C-6-R | GAGCTATTGCTTGTCCACCGAC |  |
| qCpPYL4-F | CATACAGTCGATCCCAACCAGTG |  |
| qCpPYL4-R | CCACTATCACGTTGCAGGTCTTG |  |
| qCpCYP707A1-F | GAACATCCCAGGAACTACGTACCAG |  |
| qCpCYP707A1-R | ATTTGATCGTCACTGAGCAGCTC |  |
| qCpSAPK7-F | AGATTTAGCGAGGATGAGGCG |  |
| qCpSAPK7-R | CCGACGACTTGGAATAACCG |  |
| qCpNCED3-F | AATCGGGTCTGAGTCATCGTG |  |
| qCpNCED3-R | TTCTGGCGTGAGACAAGCG |  |
| qCpSRK2E-F | TGATGATGACATGGACGACTTAGA |  |
| qCpSRK2E-R | CAACCTCTTTACACTTAGCCCAC |  |
| qCpABI3-F | ATCTTCTTTCGATGCCACATTC |  |
| qCpABI3-R | GCTACTCACATCGCTTTGTTTTAGT |  |
| qCpABI5-1-F | TCAAGAACCGTGAATCAGCAG |  |
| qCpABI5-1-R | GGCTATTGCCATCATTGCTTC |  |
| qCpABI5-2-F | CAGATCCAGCATTCAGAAGTAACC |  |
| qCpABI5-2-R | ATTCCCTTCGCTCGTTTGA |  |
| qCpABI5-3-F | TGAAGGTTTCTGGTGCTTGTTG |  |
| qCpABI5-3-R | TGGACCTCATCAAGGGTAAGATT |  |
| q*CpIAA16*-F | CATCCTTATCTTCATATGTGGGCAC |  |
| q*CpIAA16*-R | AGATGTTCAGTTCCTTCACCAGTGG |  |
| q*CpCWINV1*-F | GGAAGTGATAGCCAGTTCTGTAATT |  |
| q*CpCWINV1*-R | CAACCTAAGAGACGACTCGACG |  |
| q*AtSOC1*-F | AGCTGCAGAAAACGAGAAGC |  |
| q*AtSOC1*-R | TGAAGAACAAGGTAACCCAATG |  |
| q*AtLFY*-F | CTCTATTTGGTATGTTCCAACAAAG |  |
| q*AtLFY*-R | CTAATACCGCCAACTAAAGCC |  |
| q*AtAP1*-F | TAGGGCTCAACAGGAGCAGT |  |
| q*AtAP1*-R | CAGCCAAGGTTGCAGTTGTA |  |
| q*AtSEP3*-F | CCAACTCTATTTGAATCTTTCTCAC |  |
| q*AtSEP3*-R | ACAAGACAGAAAACATGAGAGAGGT |  |
| q*AtActin*-F | CTTCGTCTTCCACTTCAG |  |
| q*AtActin*-R | ATCATACCAGTCTCAACAC |  |
| *CpFT1*-F | CGGGATCCATGCCCAGGGAAAGAGATCCT | Full-length isolation of CDS sequence and vector construction |
| *CpFT1*-R | GGAATTCTGGAACTGCTGTTGGACCTAT |  |

Note: Underline sequences are cleavage sites of restriction enzyme.
