# Supplementary material for: Integrated transcriptome and proteome analysis provides insight into chilling-induced dormancy breaking in Chimonanthus praecox
Source: Hortic Res. 2020 Dec 1;7:198. doi: 10.1038/s41438-020-00421-x (PMC7704649; doi:10.1038/s41438-020-00421-x)
Supplement: Supplementary file 12 — Supplementary Figures [file 41438_2020_421_MOESM12_ESM.docx]

**Supplemental Material**

**
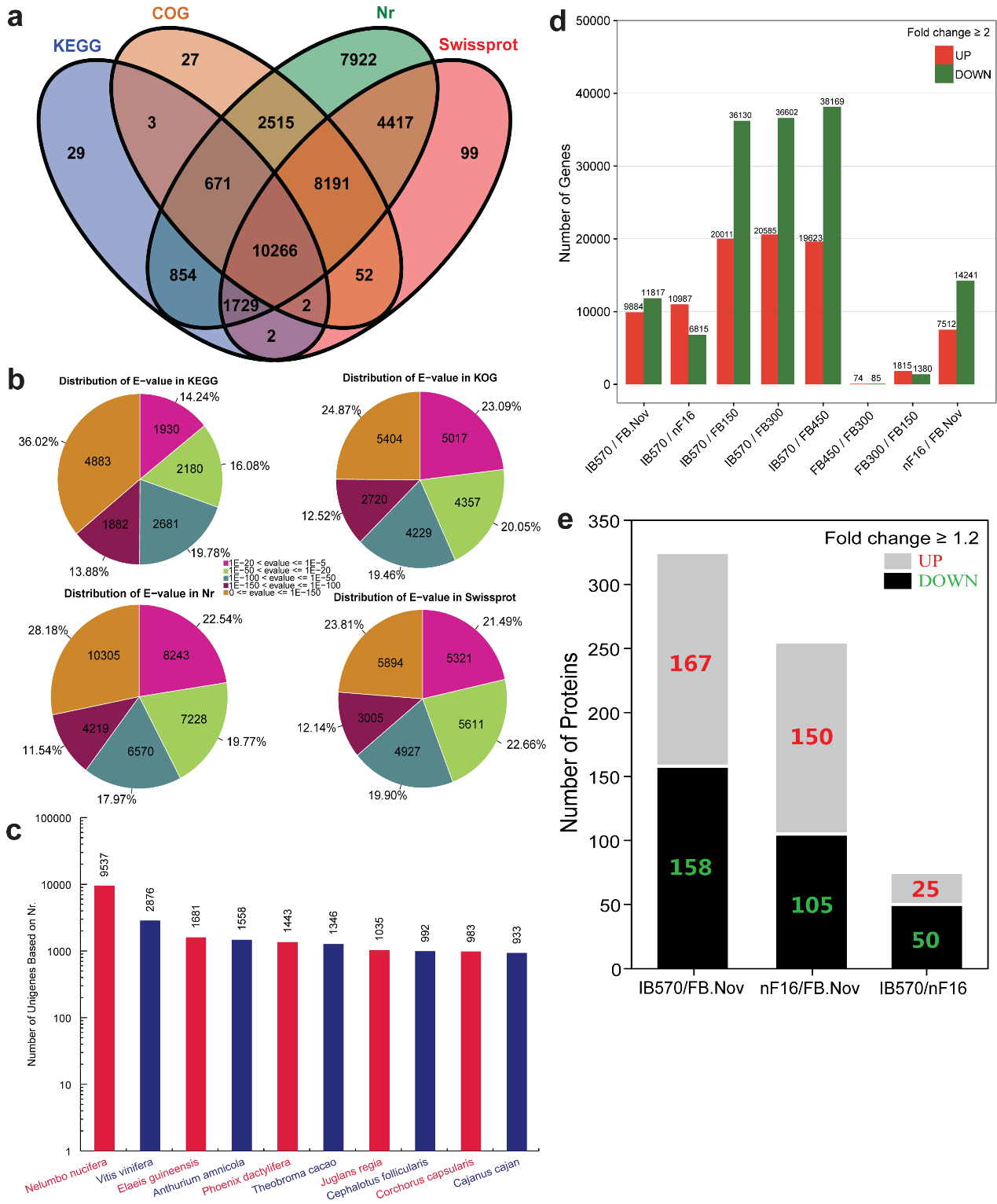
**

**Supplementary Fig. S1 Annotation of assembled *C. praecox* unigenes and differentially expressed genes (DEGs) and proteins. a** The numbers of the 36779 unigenes annotated by different databases, including KEGG, KOG, Nr and SwissProt, are shown in a Venn diagram. **b** Distribution of E-values in the KEGG, KOG, Nr and SwissProt databases. **c** Identification of the transcripts of other plant species homologous to the annotated unigenes of *C*. *praecox*. **d**-**e** DEGs and proteins in FB and/or IB comparisons IB570/FB.Nov, IB570/nF16, IB570/FB150, IB570/FB300, IB570/FB450, FB450/FB300, FB300/FB150 and nF16/FB.Nov.

**
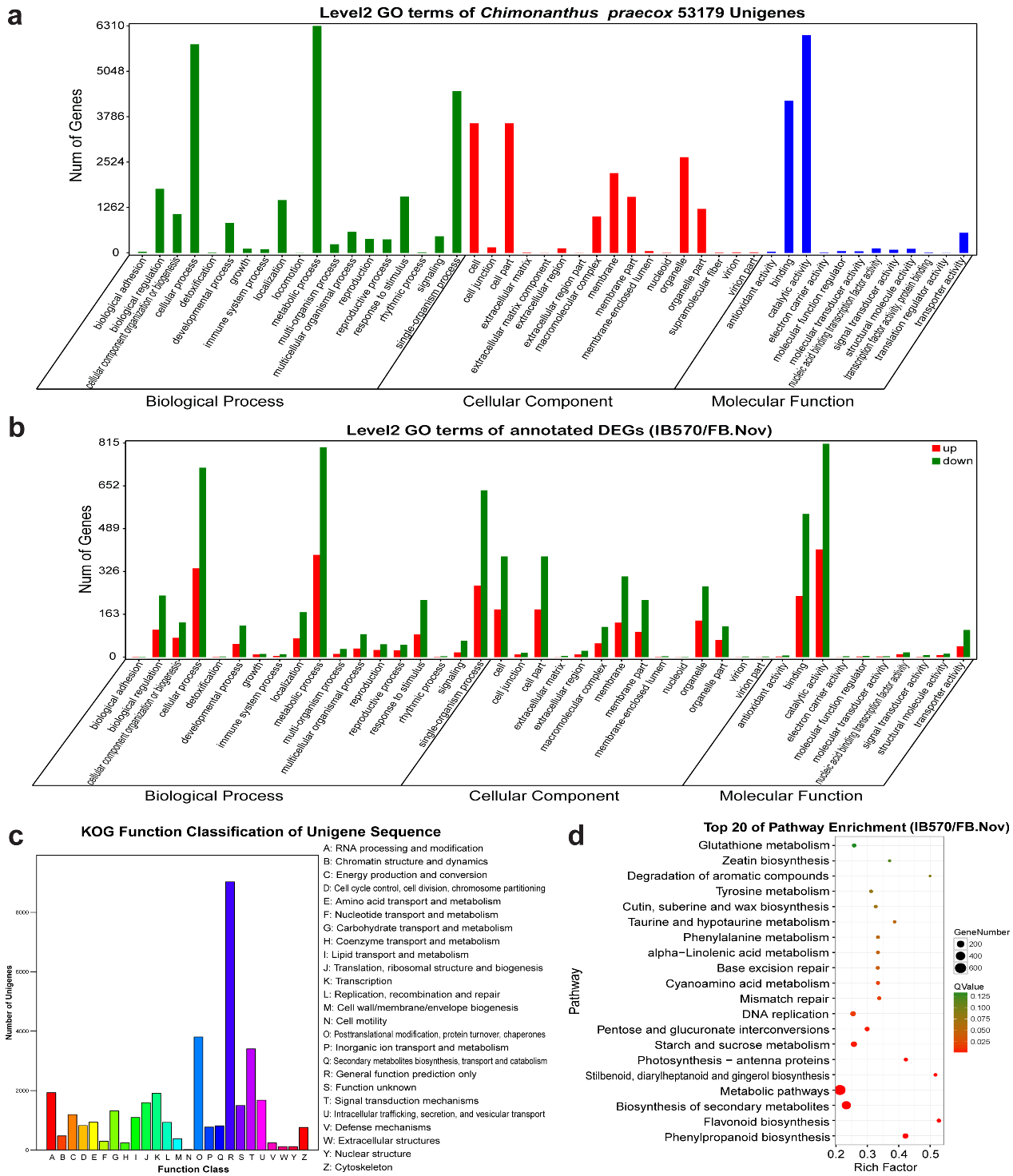
**

**Supplementary Fig. S2 GO, KOG and KEGG analysis of DEGs. a**-**b** GO assignments for the 18 sample transcriptomes and up-/downregulated DEGs in IB570/FB.Nov of wintersweet. The results are summarized under three main GO categories: BP, CC and MF. The y-axis indicates the number of genes in the same category. Up- and downregulated DEGs in IB570/FB.Nov. **c** KOG functional classification for the transcriptome of wintersweet. From a total of 100,553 de novo assembled transcripts, 35,354 transcripts with significant homologies in the KOG database (E-value≤1.0 E-5) were classified into 25 KOG categories. **d** Top 20 enriched pathways in IB570/FB.Nov. The top three KEGG pathway enrichments in IB570/FB.Nov based on the Q-value were as follows: phenylpropanoid biosynthesis (ko00940; rich factor, 88/209; Qvalue, 7.17E-14; the same below), flavonoid biosynthesis (ko00941; 28/53; 9.43E-07), and biosynthesis of secondary metabolites (ko01110; 357/1534; 1.29E-06), followed by starch and sucrose metabolism (ko00500; 96/373; 3.16E-03), plant hormone signal transduction (ko04075; 73/326; 1.64E-01) and the circadian rhythm-plant pathway (ko04712; 15/72; 0.16), ranking 7^th^, 26^th^ and 52^th^, respectively, among a total of 128 pathways. In the case of IB570/nF16, they were pentose and glucuronate interconversions (ko00040; rich factor, 52/164; 3.11E-07), starch and sucrose metabolism (ko00500; 79/373; 4.85E-05), phenylpropanoid biosynthesis (ko00940; 49/209; 4.39E-03) and flavonoid biosynthesis (ko00941; 18/53; 5.05E-03).

**
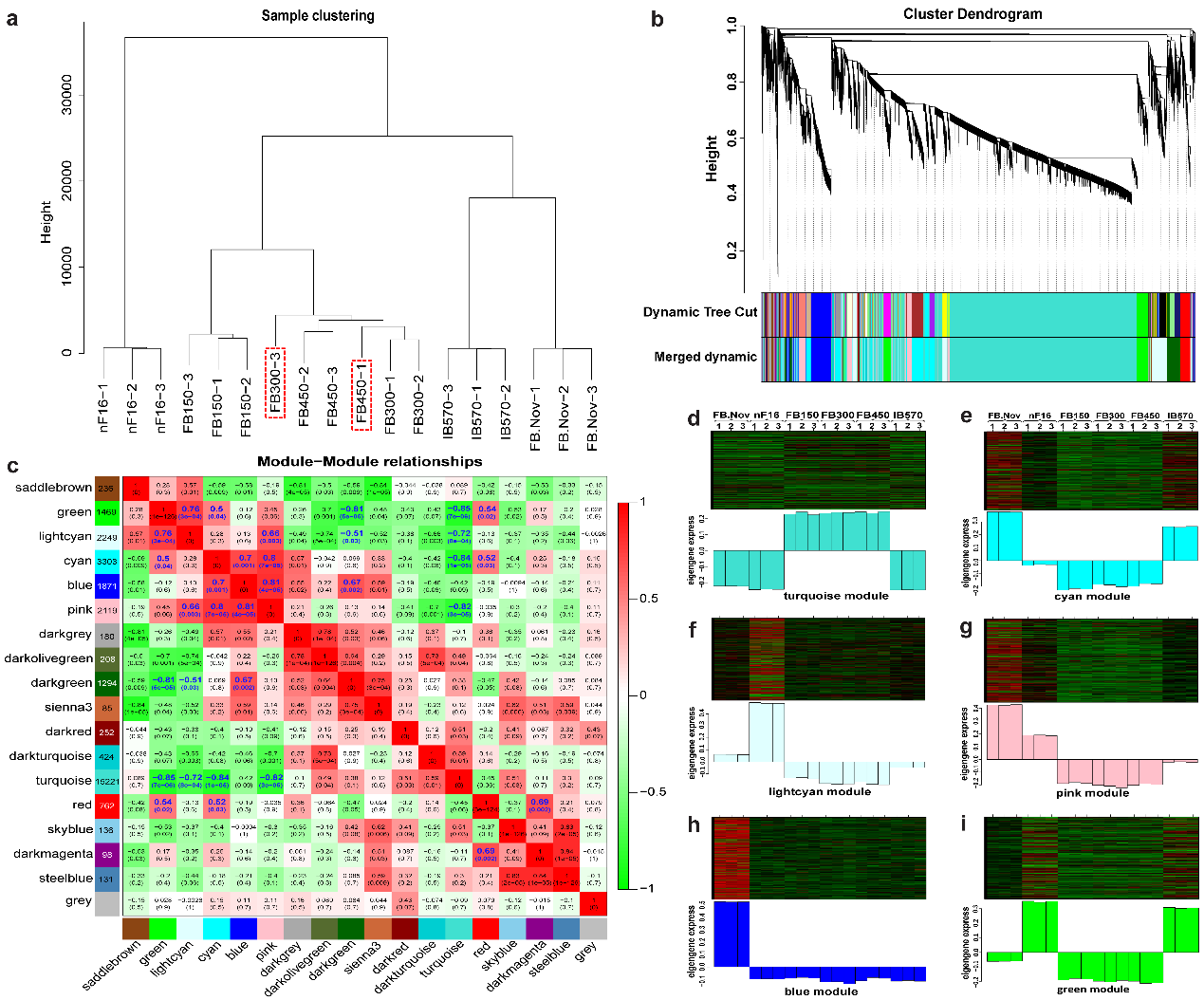
**

**Supplementary Fig. S3 Sample cluster, hierarchical cluster, module-module relationship and eigen gene expression patterns based on WGCNA. a** Sample clustering. Red, dashed rectangles are placed around outlying libraries. **b** Hierarchical cluster tree. **c** Module-module relationship. **d**-**i** Eigen gene expression pattern of turquoise (**d**), cyan (**e**), light cyan (**f**), pink (**g**), blue (**h**) and green (**i**) modules.

**
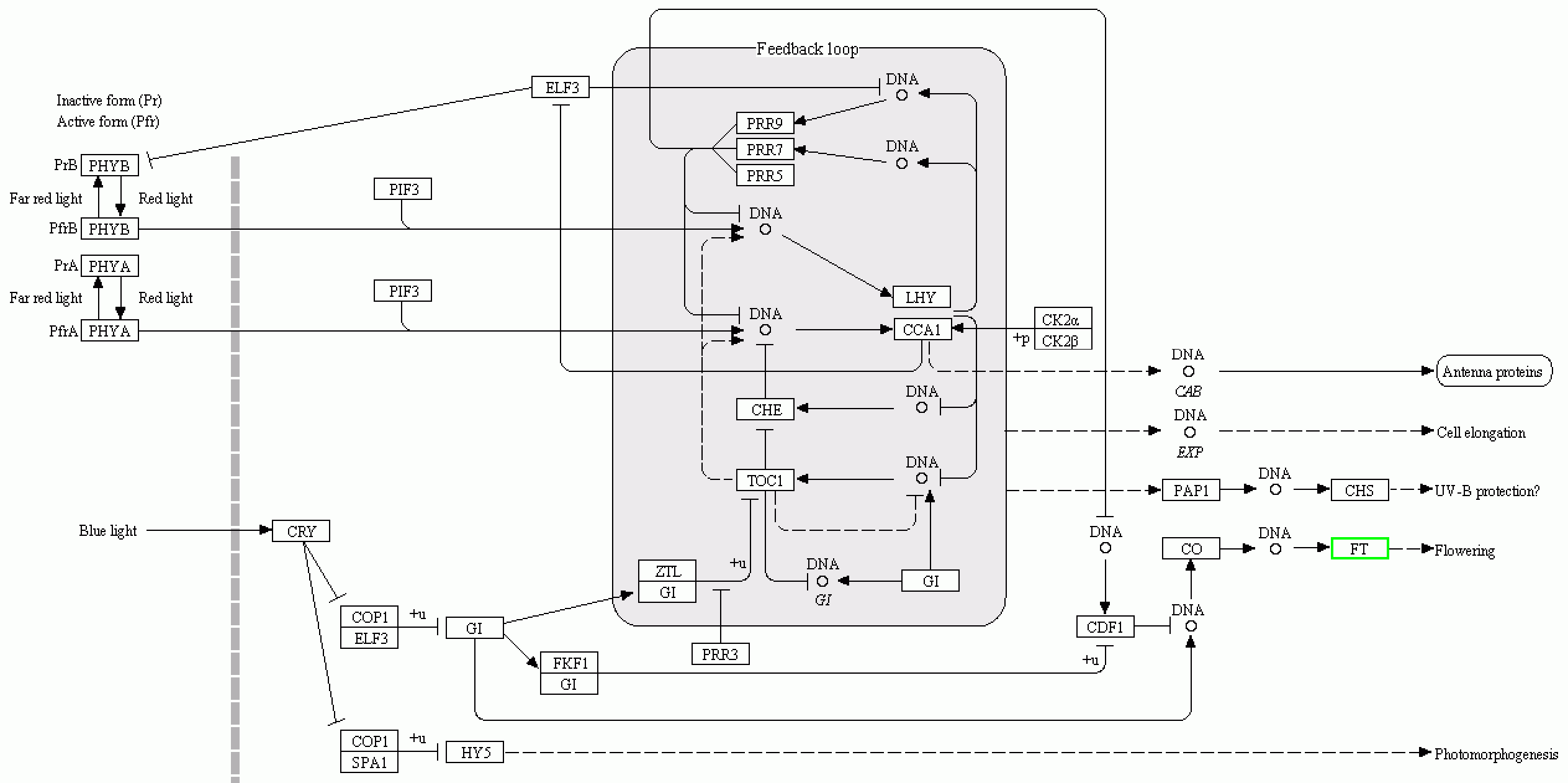
**

**Supplementary Fig. S4 Circadian rhythm-plant pathway enrichment from the 1628 intersection in Fig. 4d based on KEGG.** Unigene0043357 *FT1* was significantly enriched.


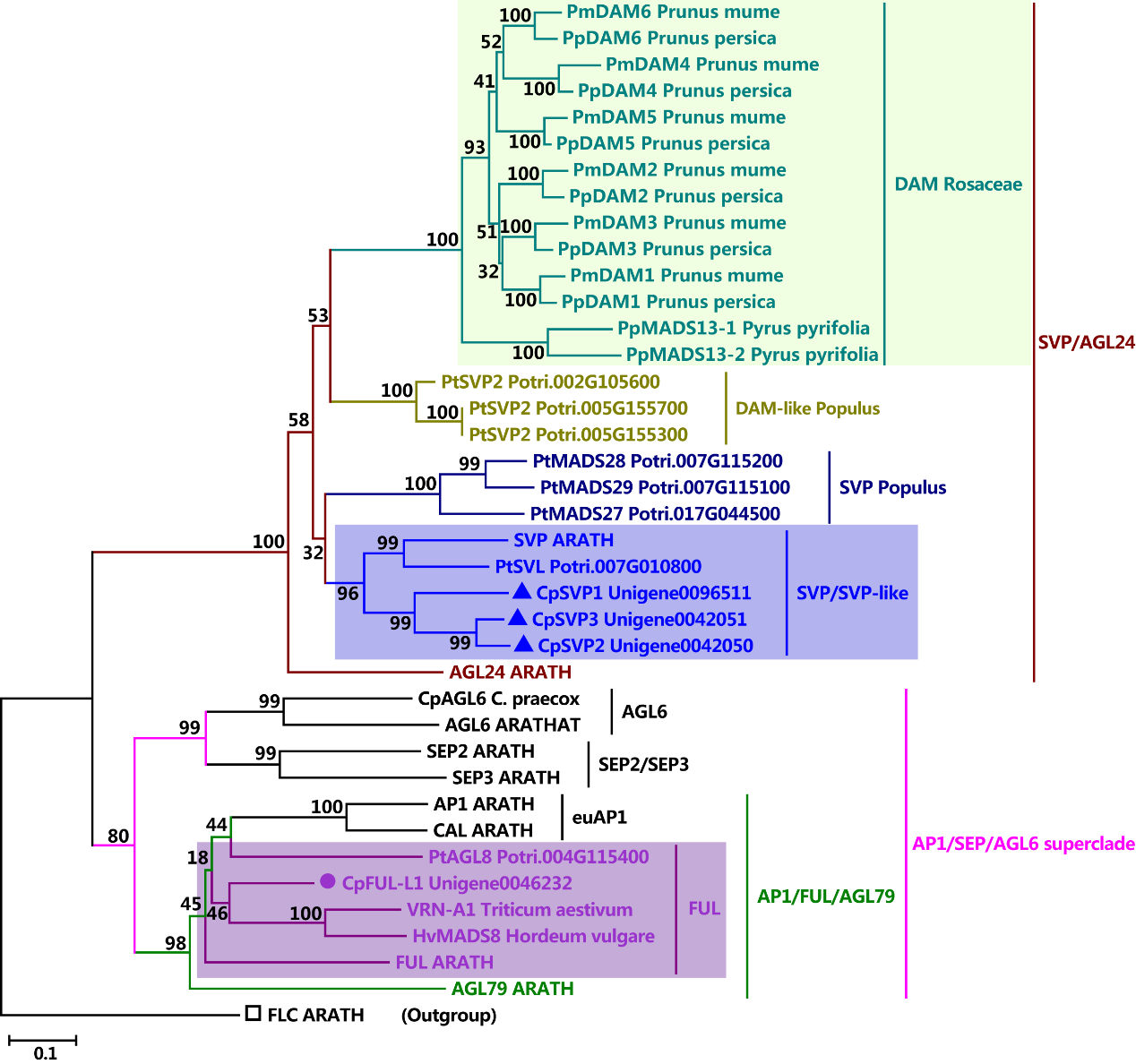


**Supplementary Fig. S5 Phylogenetic tree of DEGs from STMADS11 and AP1/SEP/AGL6 superclades.**

**
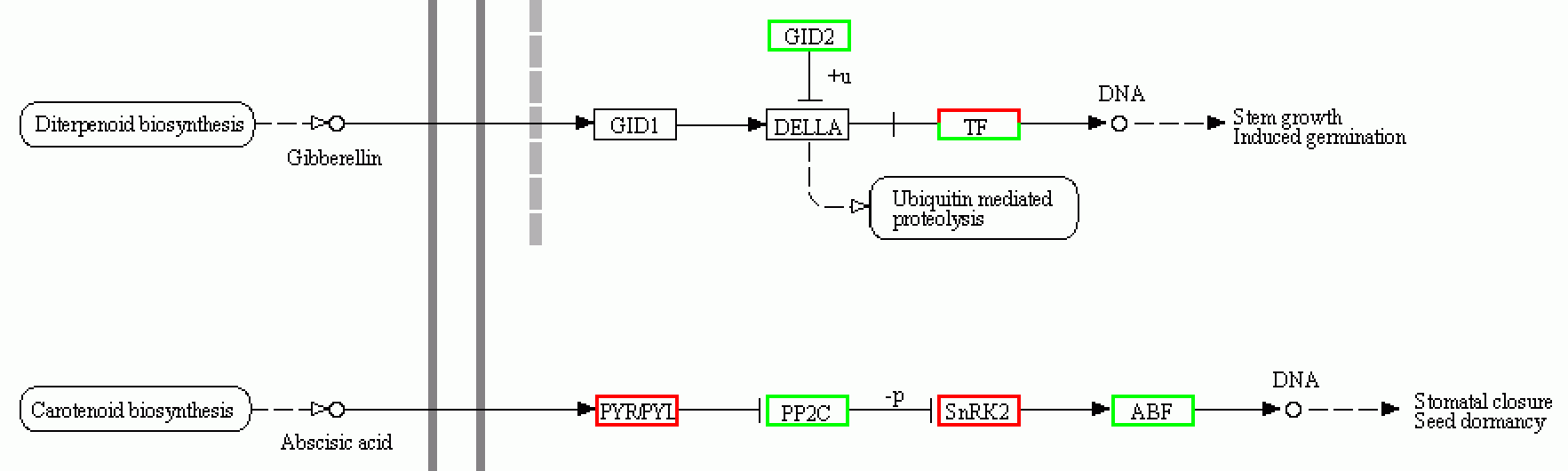
**

**Supplementary Fig. S6 Plant hormone signal transduction enrichment based on KEGG pathway analysis of IB570/FB.Nov.**

**
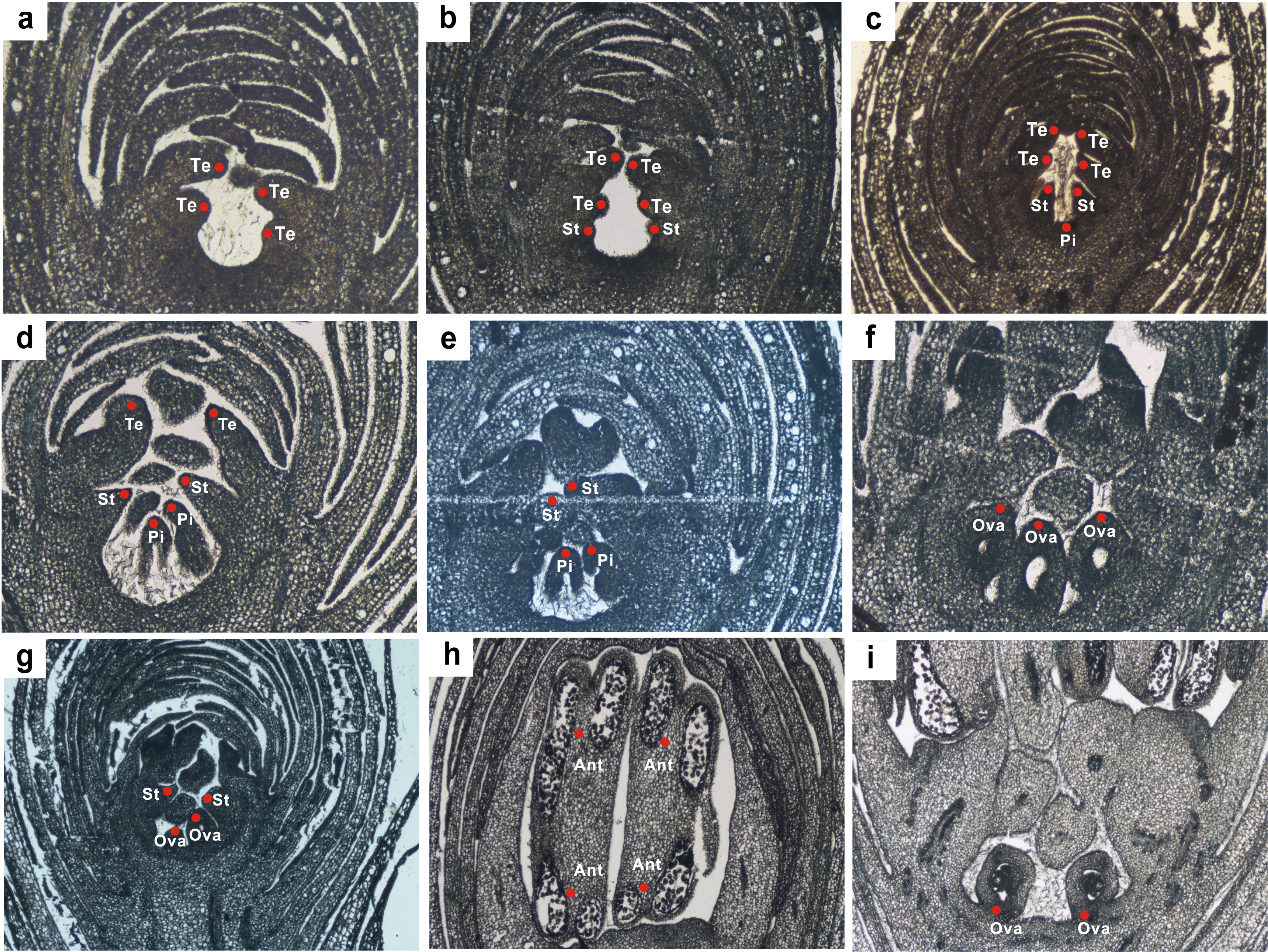
**

**Supplementary Fig. S7 The differentiation of *C. praecox* flower buds in different periods.** Longitudinal section of flower buds at different primordia differentiation stages: TDS, tepal differentiation stage (**a**); SDS, stamen differentiation stage (**b**); PDS, pistil differentiation stage (**c**-**e**); Ova, ovary differentiation stage (**f**-**g**); Ant, anther development (**h**); differentiation completed (**i**). Te, St, Pi, Ova and Ant represent tepal, ovary and anther, respectively.

Supplementary Table S1 List of primer pairs used in this study.

Supplementary Table S2 GO annotations of 53179 unigenes in *C. praecox*. Related to Fig. S2a.

Supplementary Table S3 GO annotations in IB570/FB.Nov & IB570/nF16. Related to Fig. S2b.

Supplementary Table S4 GO enrichment (biological process) in IB570/FB.Nov & IB570/nF16 (BP).

Supplementary Table S5 KOG classification and annotations of 35,354 unigenes. Related to Fig. S2c.

Supplementary Table S6 KEGG pathways of all unigenes.

Supplementary Table S7 KEGG pathway in IB570/FB.Nov & IB570/nF16. Related to Fig. S2d.

Supplementary Table S8 WGCNA. Fig. 2c-d, S3.

Supplementary Table S9 Seventy-six out of 161 of 701 selected DEGs used for the coexpression network in Cytoscape. Related to Fig. 3.

Supplementary Table S10 461 candidate DEGs and 26 DEGs for the heatmap. Related to Fig. 4e, 7b.

Supplementary Table S11 KOG classification and annotations of TMT. Related to Fig. 5c.
